# Supplementary material for: The NICU Cuddler Curriculum: A Service-Learning Curriculum for Preclinical Medical Students in the Neonatal Intensive Care Unit
Source: MedEdPORTAL. 2021 Jan 12;17:11069. doi: 10.15766/mep_2374-8265.11069 (PMC7809928; doi:10.15766/mep_2374-8265.11069)
Supplement: Supplementary file 1 — Course Description.docxParticipant Application.docxOrientation Outline.docxOrientation Presentation.pptxNeonatal Abstinence Syndrome.pptxDevelopmental Care in the NICU.pptxParent Note Cards.docxPatient Log.docxAnonymous Concerns.docxStudent Survey.docxThird- and Fourth-Year Student Survey.docxEmail to Nursing Staff.docx [file mep_2374-8265.11069-s001.zip › _Educational_Summary_Report_11069.pdf]

MedEdPORTAL® The AAMC Journal of Teaching and Learning Resources

Original Publication Open Access

The NICU Cuddler Curriculum: A Service-Learning Curriculum for Preclinical Medical Students in the Neonatal Intensive Care Unit

Elena Insley*, Kathleen Tedesco, Ethan A. Litman, Nikitha Mangalapally, MS, Casey Gicewicz, MSN, RN, Meredith L. Monaco-Brown, MD

*Corresponding author: eminsley@gmail.com

Abstract

Introduction: The neonatal intensive care unit (NICU) is often seen as off-limits by preclinical medical students. The NICU cuddler curriculum is a service-learning curriculum that invited preclinical medical students into the NICU to engage with and learn from one of the hospital's most vulnerable populations: neonates. The purpose of this preclinical experience was to provide students with exposure to the NICU and an opportunity to engage with babies, families, and the NICU staff, in order to improve students' clinical and communication skills. **Methods:** First- and second- year medical students applied and were selected for participation. Participants cuddled neonates in the NICU for at least 10 hours, attended didactic sessions relevant to neonatal care, and debriefed with an attending each semester. The curriculum was evaluated via qualitative analysis and postparticipation surveys. **Results:** To date, a total of 73 students have participated in the NICU cuddler curriculum. Qualitative analysis revealed students felt included in patient care, empowered in their understanding of the social determinants of health, and useful in their role. A postsurvey of clinical medical students following participation revealed the sustained impact of this program. **Discussion:** This service-learning curriculum for preclinical medical students has the potential to enhance student understanding of the social determinants of health, increase exposure to the NICU, and promote interprofessional collaboration, ultimately increasing preparedness of students for their clinical years.

Keywords
Service-Learning, Preclinical, NICU, Professionalism, Neonatal-Perinatal Medicine, Clinical Teaching/Bedside Teaching

Educational Objectives

By the end of this activity, learners will be able to:

1. Discuss risk factors, including clinical conditions and social determinants of health, that result in a neonatal intensive care unit (NICU) admission.
2. Demonstrate interpersonal skills such as working with nurses and other staff and examine the benefits of healthy interdisciplinary teamwork.
3. Practice basic skills used to comfort newborns and aid in their development.
4. Identify social and emotional stressors faced by families of infants hospitalized in the NICU.

Citation:
Insley E, Tedesco K, Litman EA, Mangalapally N, Gicewicz C, Monaco-Brown ML. The NICU cuddler curriculum: a service-learning curriculum for preclinical medical students in the neonatal intensive care unit. *MedEdPORTAL*. 2021;17:11069.
https://doi.org/10.15766/mep_2374-8265.11069

Introduction

Service learning, as dictated by the Liaison Committee on Medical Education,[1] is a unique form of structured community service which combines classroom-based learning with community relationship development.[2,3] Through service-learning students are able to use real-world experiences in their community to enhance their classroom-based education.[4] This type of educational model helps medical students better understand the relationship between biologic and nonbiologic social determinants of health[5] in their community. Service-learning curricula have been shown to improve student preparedness for clinical duties and help students understand barriers to health care access, illuminate disparities in health care, improve cultural competency, and develop professionalism.[6,7] These were all achieved within an effective service-learning curriculum by Shakartzi et al[8] which empowered preclinical students with the ability to offer labor support to pregnant women in underserved communities.

Currently, undergraduate medical education provides limited experiences in the critical care setting, particularly the neonatal

Copyright © 2021 Insley et al. This is an open-access publication distributed under the terms of the Creative Commons Attribution license.

intensive care unit (NICU). As the number of residents interested in pursuing adult critical care fellowships decline; some suggest that decreased ICU exposure during undergraduate medical education has played a role.[9-12] In addition, neonatal-perinatal fellowship match data reveal a decrease in unmatched applicants (21 to 9) and an increase in unmatched programs (17 to 25) from 2016-2020, which may represent waning interest in applicants compared to number of positions.[13] To help increase exposure to this field we established the NICU cuddler curriculum which allowed preclinical immersion in the NICU accompanied with structured education sessions allowing students a safe opportunity to increase familiarity with the clinical and critical care setting, and better understand the social determinants of health through hands-on experiences in a clinical NICU setting.

This curriculum was also developed to address entrustable professional activity (EPA) 9—collaborate as a member of an interprofessional team—by providing training and opportunities to begin developing these skills in the NICU. The AAMC outlines Core EPAs for Entering Residency which are 13 distinct “units of observable, measurable professional practice requiring integration of competencies” assessed together to determine readiness for residency.[14] Creating opportunities for medical students to practice and hone their interpersonal skills and ability to work effectively on an interdisciplinary team is essential for a successful transition out of medical school and into residency and beyond.

The NICU cuddler curriculum was established to increase preclinical medical students’ awareness and understanding about societal and medical implications in a variety of NICU scenarios, encourage interprofessional development between medical students and clinical staff, and hone basic hands-on skills in the NICU. This curriculum allowed students to practice these skills in a setting that was also beneficial to vulnerable patients and families.

Methods

Curriculum Overview

The NICU cuddler curriculum was a preclinical elective service-learning curriculum of NICU-themed lectures and NICU exposure via clinical meeting attendance and baby-holding, or cuddling. This formal and sustainable program was developed by four first- and second-year medical students in conjunction with the NICU nursing administration and an attending physician from the department of neonatology.

Application

Preclinical medical students were notified about the NICU cuddler curriculum via the institution’s service-learning department, which advertises programs available to students by providing course descriptions for each program (Appendix A) as well as hosting a biannual service-learning fair. All first- and second-year medical students received a student-designed short answer application utilizing Google Forms (Appendix B) in both semesters. The two student leaders were responsible for selecting participants based on their demonstrated interest in the short answer section of the application. Student leaders also valued diversity in school year, gender, and specialty interest when selecting participants. There was a limit of 16 students chosen per semester. This number was selected because it was deemed a manageable number for the student leaders to organize, and it maximized contact hours for participants.

Orientation and Training

Each incoming group attended a 2- to 3-hour orientation session (Appendix C). Orientation began in a conference room with a PowerPoint presentation (Appendix D). The classroom portion of the orientation included education in basic neonatal clinical care, standard NICU operating procedures, and professional communication styles. Following the PowerPoint presentation and classroom-based discussion, students were taken into the NICU for the hands-on portion of the training. Students received a tour of the NICU, were acclimated to patient rooms, and were able to practice newborn holding skills with patients under the supervision of a NICU attending or nurse manager. All hands-on patient training was only with patients who had been previously vetted as medically appropriate to cuddle, and with consent from the families whenever possible, and not with any family who had opted out of the program. Students also received training on standard precautions and transmission-based precautions, as well as handling instructions for basic NICU equipment, such as an isolette. This training utilized bedside nurses, which helped familiarize new students and the nursing staff with the program.

Clinical NICU Meetings

NICU cuddler students were invited to recurring hospital clinical conferences. These lectures included a bimonthly maternal-fetal medicine conference held in conjunction with OBGYN, a monthly NICU ethics conference, and a weekly palliative care conference specifically for NICU patients. They were also welcomed to shadow medical rounds. These were established events held regularly at our institution where cuddlers were encouraged to attend as their schedule allowed, but were not mandatory for participation. These experiences further developed the

Copyright © 2021 Insley et al. This is an open-access publication distributed under the terms of the Creative Commons Attribution license.

interdisciplinary nature of this program as various specialties and professionals attended, and highlighted the unique clinical, social, and ethical content experienced in the NICU.

NICU Lecture Series

While the hands-on portion of the program was the most significant in terms of time, commitment, and training, there was an associated NICU lecture series to accompany the clinical experience. Once per semester, participants in the program attend a 1.5-hour lecture on a relevant NICU topic led by a neonatologist or related faculty. The topics were chosen based on student interest in conditions or issues they were exposed to during cuddling sessions. Two topics presented were neonatal abstinence syndrome (NAS) and neonatal development (Appendices E and F).

Cuddling Sessions

During the hands-on portion of orientation, the students were taught how to introduce themselves to the nurse and ask relevant questions about the patient. Then they were shown how to hold a baby, including providing head support in different upright and supine positions. It was demonstrated how to manage associated wires and tubing, and what to do if the patient had a monitor alarm, or was clinically concerning to the cuddler. The students were given suggestions about what to do with the baby while holding, such as softly talking, singing, reading, or sitting quietly. They learned that it is ok to ask to transfer a baby back into bed if they became sleepy or physically uncomfortable while cuddling, or if a baby's crying had created significant stress for the cuddler. They also role-played introducing themselves to a parent who enters during a cuddling session.

In working closely with the NICU staff, 2-hour shifts were organized to occur 3 days each week. During this time, medical students who were selected for, oriented to, and trained in the program would be available in the NICU to hold neonates. Two students were assigned to each shift and were encouraged to cuddle up to three neonates during their 2-hour shift depending on infant feeding/care schedules, a need to console crying babies, and/or parent availability. As cuddlers were not expected or allowed to feed the patients, their holding time was sometimes limited by the timing of the next feed.

At the beginning of each shift, two students entered the NICU and wrote their names on the staffing bulletin board as cuddlers that were present on the floor. The cuddlers went to each nurse's station, introduced themselves, and asked which patients were in need of cuddling. Families were provided with a brochure that contained detailed information about the program and how to opt out if desired. Nurses selected newborns to be held based on the neonates' estimated length of stay, presence of bedside family members, clinical stability, and overall temperament. Neonates were ineligible to participate if they were intubated, weighed less than 1,000 grams, or had an anticipated length of stay less than 48 hours.

Once a patient was identified, the student would perform proper hand hygiene, don appropriate personal protective equipment, and transfer the patient from the bed into proper position with the help of the nurse. Students were equipped with the nursing call bell in case assistance was needed. Students often utilized the books in patients' rooms to read to babies while cuddling. After cuddling for approximately 1 hour, students would contact the nurse, and together they would safely transfer the baby back to bed. Before leaving the patient's room, the student wrote a notecard (Appendix G) to the patient's parents to let them know their baby was cuddled. At the end of the shift, the students recorded their cuddling sessions with each neonate in a log sheet detailing the date, medical records number, and whether a postcard was left (Appendix H).

Medical Student Support

Students were autonomous during their shifts and worked together in pairs to see patients, interact with the nursing staff and faculty, as well as interface with parents on occasion. Students were involved in the critical care setting with high acuity patients; as a result, it was necessary to ensure they had support throughout their participation. The neonatologist and nurse manager, who served as mentors for the program, were accessible to students via email and on the unit. Student leaders utilized an anonymous concerns form that allowed student volunteers to submit experiences, questions, or concerns at any time (Appendix I). The form offered an option to include the student's name/contact information if they wished to discuss their submission further. At the end of both semesters, faculty led a mandatory reflective debriefing for all participants where we discussed both specific and general impressions and concerns from the semester.

Assessment

Real-time assessment of the program occurred during each end-of-semester debriefing mentioned above where students were encouraged to voice concerns, suggest improvements, and reflect on positive experiences and growth throughout the semester. These sessions allowed for direct feedback and discussion between program leadership and participants and ensured that students felt safe and supported throughout their involvement in the program.

Copyright © 2021 Insley et al. This is an open-access publication distributed under the terms of the Creative Commons Attribution license.

We formally assessed this program at the end of its second year. Anonymous supplemental surveys were sent by email (Qualtrics) to medical students (Appendix J). These surveys were then analyzed via qualitative analysis to assess for achievement of learning objectives, areas for growth and improvement within the program, and personal growth of participants after involvement in the program. Finally, we anonymously surveyed third- and fourth-year medical students who participated in the program during their first and/or second year of medical school (Appendix K). This survey assessed the lasting impact of the program, continued achievement of the learning objectives, and influence of the program on clinical skills and experiences in the third and fourth years of medical school.

All NICU nurses were informed of the program via email (Appendix L) prior to initiation of the program and were invited to provide operational feedback and suggestions.

Results

Most students were accepted during their first year and continued on with the program through the completion of their second year. The NICU cuddler curriculum was initially rolled out with five student volunteers in order to establish any barriers or challenges. They participated in 2-hour shifts. The following semester, applications were distributed as described; over 60 students responded and 10 were chosen (five first-year medical students, five second-year medical students) in addition to the original five (Table 1). There were a total of 15 student participants during the first year. The program concluded its third full year as an established service-learning curriculum at Albany Medical College in Spring 2019. By the conclusion of the 2019 academic year the program had reviewed over 130 applications and accepted 73 members. The 2018-2019 academic year saw the greatest number of student participants for a total of 55. From 2017-2019, over 350 cuddling encounters were recorded.

Table 1. The Neonatal Intensive Care Unit Cuddler Curriculum Participation Growth Over Time

	New Participants		
Timeframe	Total	First-Year *N* (%)	Total Participants
Fall 2016	5	3 (60)	5
Spring 2017	10	7 (70)	15
2016-2017 total	15	10 (67)	15
Fall 2017	15	12 (80)	25
Spring 2018	13	13 (100)	38
2017-2018 total	28	25 (66)	38
Fall 2018	16	11 (69)	41
Spring 2019	14	14 (100)	55
2018-2019 total	30	25 (83)	55
2016-2019 total		60 (82)	73

We anonymously surveyed the NICU cuddlers following completion of the second year of the program. A qualitative analysis was then performed on short answer survey results and revealed a number of recurring themes in students' experiences including integration into the health care team, exposure to the ICU setting, and understanding social determinants of health. The results of this survey were categorized by theme which correlated to all four of the program's educational objectives (Table 2).

Third- and fourth-year medical students who participated in the NICU cuddler curriculum during their preclinical years were surveyed to assess the accomplishment of the learning objectives of the curriculum. We sent the survey to 37 students, and 27 (73%) responded. As a result of their participation in the curriculum, most students (78%) were more confident in recognizing and discussing risk factors for NICU admission, such as social determinants of health and clinical conditions that result in a NICU admission (question 1). A majority of students (63%) felt more competent and comfortable working with an interprofessional team of nurses and other staff, and were able to discuss the benefits of such skills (question 2). All students (100%) felt increased confidence in basic cuddling skills such as holding neonates and transferring them to and from other providers or caregivers (question 3), and 93% felt the curriculum increased their understanding of the social and emotional stressors faced by families of NICU patients (question 4). Finally, the survey showed that 63% of respondents felt the NICU cuddler curriculum provided them with transferable skills that they employed during their third- and fourth-year experiences in the clinical setting (question 5). Although in some of these responses we saw only two-thirds respond positively, 89% of all respondents responded positively to at least three of the five questions mentioned above.

The same third- and fourth-year students were also asked in what ways, if any, the NICU cuddler curriculum prepared them better for clinical rotations. While there were over 14 distinct answers to this question, the majority of students reported that participation in the program made them feel more comfortable in patient rooms (93%), improved their communication with nursing staff (82%), and helped them better understand complex family and social dynamics (82%).

Discussion

The NICU cuddler curriculum was created out of the desire of preclinical medical students to experience the NICU. Through multidisciplinary collaboration, it became a program that

Copyright © 2021 Insley et al. This is an open-access publication distributed under the terms of the Creative Commons Attribution license.

Table 2. Excerpts From Medical Student Participant Surveys Categorized by Educational Objective

Educational Objective	Student Excerpts
1: Discuss risk factors, including clinical conditions and social determinants of health, that result in a NICU admission	"The ...program is about so much more than cuddling. I did not anticipate the exposure I would get to learning about the medical issues surrounding the babies, the difficulties in their care ..."<br>"I also learned about NAS babies and how they recover and cope."<br>"We get the chance to serve a vulnerable population ...we learn about the social determinants of health and how dynamic family relationships can impact the baby"
2: Demonstrate interpersonal skills and examine the benefits of healthy interdisciplinary teamwork.	"I learned how to interact with support staff on medical units."<br>"I learned how to work as a team with the NICU staff and clinical team."<br>"Holding her in sterile clothes and working with the nurse to ensure a successful feed was a moment that blended the clinical aspect of the program with the patient service portion. I truly felt that in this moment, the baby needed me as did the nurse. I felt a valuable member of the team and a positive moment in this baby's otherwise difficult life thus far."
3: Practice basic skills used to comfort newborns and aid in their development.	" ...The baby needed me as did the nurse. I felt a valuable member of the team and a positive moment in this baby's otherwise difficult life thus far."<br>"I learned how to cuddle delicate babies, how to interact with support staff on units, how the NICU runs efficiently, and about various medical conditions babies experience. It was also useful because I could immediately see the effects of my service—a happy baby!"
4: Identify social and emotional stressors faced by families of infants in the NICU.	"I recently held a baby with NAS. As I learned more about her I became enraged...Her parents were supposed to be around to hold her and comfort her, but according to their nurse, they had snuck out...I had the opportunity to meet the parents of this baby... I was surprised that instead of anger, I was met with profound sadness...I didn't have to ask them details about their lives to see that whatever they were put up against was too much for them to overcome."<br>"I was holding a baby and speaking with a nurse about the baby's future and the prognosis of her illness. It was heartbreaking to hear about the uncertain and complicated future of the adorable baby I was holding. It was hard to hear about her less-than-ideal family life and the difficulty of her condition. I felt helpless but was incredibly grateful that the staff in the NICU was advocating for the baby and doing their absolute best for her, and it made me happy that I was doing my best with my skills to help her, too."

Abbreviations: NAS, neonatal abstinence syndrome; NICU, neonatal intensive care unit.

benefitted the students, the staff, neonates, and their families. The objectives of this curriculum sought to address goals and objectives of the service-learning curriculum at our institution as well as the AAMC's Core EPAs for Entering Residency, in particular, EPA 9: collaborate as a member of an interdisciplinary team.[14] The educational objectives outlined at the outset of the curriculum were largely met as demonstrated by both a qualitative analysis and postsurvey of clinical students. Overall, students felt more comfortable in their skills holding and maneuvering neonates, interacting with nursing and hospital staff, and understanding complex social dynamics that surround NICU admissions. This was a unique experience that allowed preclinical medical students, who often have limited patient interaction, hands-on experience in an ICU setting.

Many issues needed to be addressed when we initiated this curriculum and then sustained it. To begin, the team needed to defend the value of a curriculum with as soft a name as "The NICU Cuddler Curriculum" to the Service-Learning Committee, by demonstrating the service it provided for patients, families, and staff, and the educational opportunities it provided for the students. The case we made was convincing enough that that the group was later chosen to speak about the curriculum at an annual luncheon held by the Service-Learning Committee. In addition, the process of rolling this program out to the NICU staff and parents was carefully thought out and deliberately timed. Staff and parent buy-in was essential for allowing the students to have a variety of experiences. It was essential to include nursing management in this program from the beginning, as our nurse manager best understood both workflow concerns and also the likely responses of nurses to the program and was able to anticipate issues before they occurred.

Of course, there were limitations to this curriculum. To start, it was difficult to meet our objectives at very high rates. This was in part due to the restriction on time and effort that an elective for preclinical medical students must have in order to preserve time for their studies. In addition, the variety of cuddling experiences differed from student to student. This was a result of the wide variety of patients and nurses, as well as the fact that the cuddling experiences were largely obtained independently, due to the lack of faculty presence to regularly to help standardize each cuddler's experience. We attempted to mitigate variation in experience with a debriefing session. There, participants shared their different experiences in the group which allowed students to reflect on a larger group of experiences. One other limitation was the small number of participants, but this was intentional in order to maximize patient contact time and respect the time limitations of the medical student leaders.

There were several students who provided negative or constructive criticism via all of our assessments. The most

Copyright © 2021 Insley et al. This is an open-access publication distributed under the terms of the Creative Commons Attribution license.

common concerns early on involved needing the nurses to be better informed about the program, which was quickly addressed. Other students asked to expand the number of available cuddling times, which we did gradually. One student suggested alternate activities if there were no available babies to cuddle that shift, and another wanted more interaction with the cuddler group as a whole.

The student-initiated NICU cuddler curriculum has proved its sustainability over a 3-year period and has been one of the most sought-after service-learning programs in our institution. Future directions for the NICU cuddler curriculum include continued expansion through more participants and varied learning experiences that further develop clinical skills and critical thinking necessary for a physician. Future work is needed to further understand the impact of participation on medical student behavior and the curriculum's perceived value to our community. We intend to further assess the impact this program has on student readiness for clinical rotations as well as student career choice. This could be achieved using clerkship evaluations, which are variable, or by analyzing Step 2 clinical skills scores between participants and nonparticipants. We also hope to track our neonatal participants and their families to assess for any delayed or long-term effects of the program.

To our knowledge, this was the first curriculum that engaged medical students to serve as cuddlers for a NICU population, using a service-learning platform. The NICU cuddler curriculum has demonstrated a lasting impact on participants and durability as a curriculum during the preclinical years that enhances the medical school experience. The results of this program provided insight into the potential benefits of experiential service-based learning in an intensive-care setting during the preclinical years of physician training.

Appendices

A. Course Description.docx
B. Participant Application.docx
C. Orientation Outline.docx
D. Orientation Presentation.pptx
E. Neonatal Abstinence Syndrome.pptx
F. Developmental Care in the NICU.pptx
G. Parent Note Cards.docx
H. Patient Log.docx
I. Anonymous Concerns.docx
J. Student Survey.docx
K. Third- and Fourth-Year Student Survey.docx
L. Email to Nursing Staff.docx

All appendices are peer reviewed as integral parts of the Original Publication.

Elena Insley: Medical Student, Department of Medical Education, Albany Medical College

Kathleen Tedesco: Medical Student, Department of Medical Education, Albany Medical College

Ethan A. Litman: Medical Student, Department of Medical Education, Albany Medical College; ORCID: https://orcid.org/0000-0001-8640-3860

Nikitha Mangalapally, MS: Medical Student, Department of Medical Education, Albany Medical College

Casey Gicewicz, MSN, RN: Registered Nurse, Division of Neonatology, The Bernard and Millie Duke Children's Hospital at Albany Medical Center

Meredith L. Monaco-Brown, MD: Associate Professor, Division of Neonatology, The Bernard and Millie Duke Children's Hospital at Albany Medical Center

Disclosures
None to report.

Funding/Support
None to report.

Ethical Approval
Reported as not applicable.

References

1. 2019-2020 Functions and Structure of a Medical School. Liaison Committee on Medical Education. March 2020. Accessed March 18, 2018. http://www.lcme.org/publications/#Standards
2. Quinn SC, Gamble D, Denham A. Ethics and community-based education: balancing respect for the community with professional preparation. *Fam Community Health*. 2001;23(4):9-23. https://doi.org/10.1097/00003727-200101000-00004
3. Seifer SD. Service-learning: community-campus partnerships for health professions education. *Acad Med*. 1998;73(3):273-277. https://doi.org/10.1086/qrb.73.3.3036916
4. Dornan T, Littlewood S, Margolis SA, Scherpbier A, Spencer J, Ypinazar V. How can experience in clinical and community settings contribute to early medical education? A BEME

Copyright © 2021 Insley et al. This is an open-access publication distributed under the terms of the Creative Commons Attribution license.

systematic review. *Med Teach*. 2006;28(1):3-18. https://doi.org/10.1080/01421590500410971

5. Marmot M. Social determinants of health inequalities. *Lancet*. 2005;365(9464):1099-1104. https://doi.org/10.1016/S0140-6736(05)71146-6
6. Hunt JB, Bonham C, Jones L. Understanding the goals of service learning and community-based medical education: a systematic review. *Acad Med*. 2011;86(2):246-251. https://doi.org/10.1097/ACM.0b013e3182046481
7. Dornan T, Bundy C. What can experience add to early medical education? Consensus survey. *BMJ*. 2004;329(7470):834. https://doi.org/10.1136/bmj.329.7470.834
8. Shakartzi H, Wenren L, Fernandes S, et al. DREAM: empowering preclinical medical students with labor support. *MedEdPORTAL*. 2018;14:10665. https://doi.org/10.15766/mep_2374-8265.10665
9. Promes SB, Chudgar SM, Grochowski CO, et al. Gaps in procedural experience and competency in medical school graduates. *Acad Emerg Med*. 2009;16 Suppl 2:S58-S62. https://doi.org/10.1111/j.1553-2712.2009.00600.x
10. Fessler HE. Undergraduate medical education in critical care. *Crit Care Med*. 2012;40(11):3065-3069. https://doi.org/10.1097/CCM.0b013e31826ab360
11. O'Connor E, Moore M, Cullen W, Cantillon P. A qualitative study of undergraduate clerkships in the intensive care unit: it's a brand new world. *Perspect Med Educ*. 2017;6(3):173-181. https://doi.org/10.1007/s40037-017-0349-x
12. Stroh DA, Ray-Mazumder N, Norman JA, et al. Influencing medical student education via a voluntary shadowing program for trauma and acute care surgery. *JAMA Surg*. 2013;148(10):968-970. https://doi.org/10.1001/jamasurg.2013.363
13. *The Match: Results and Data: Specialties Matching Service: 2020 Appointment Year*. *National Resident Matching Program*; 2020:58.
14. Obeso V, Brown D, Aiyer M, et al. eds. *Core Entrustable Professional Activities for Entering Residency: Toolkits for the 13 Core EPAs*. Association of American Medical Colleges; 2017.

Received: May 2, 2020
Accepted: September 25, 2020
Published: January 12, 2021

Copyright © 2021 Insley et al. This is an open-access publication distributed under the terms of the Creative Commons Attribution license.
