# Supplementary material for: The NICU Cuddler Curriculum: A Service-Learning Curriculum for Preclinical Medical Students in the Neonatal Intensive Care Unit
Source: MedEdPORTAL. 2021 Jan 12;17:11069. doi: 10.15766/mep_2374-8265.11069 (PMC7809928; doi:10.15766/mep_2374-8265.11069)
Supplement: Supplementary file 1 — Course Description.docxParticipant Application.docxOrientation Outline.docxOrientation Presentation.pptxNeonatal Abstinence Syndrome.pptxDevelopmental Care in the NICU.pptxParent Note Cards.docxPatient Log.docxAnonymous Concerns.docxStudent Survey.docxThird- and Fourth-Year Student Survey.docxEmail to Nursing Staff.docx [file mep_2374-8265.11069-s001.zip › A. Course Description.docx]

NICU Cuddlers

Neonatal Intensive Care Unit/Division of Community Outreach and Medical

Education

Faculty:

[Advisor names]

Program Description:

The Neonatal Intensive Care Unit (NICU) provides life-sustaining support to newborns. It is well established that early and frequent human contact for neonates improves long-term outcomes, reduces stress levels and pain,

and shortens the duration of hospital stays. A medical student-run organization that increases human contact for neonates improves the long-term health of patients and enhances undergraduate medical education.

The curriculum seeks to:

• Expose medical students to the neonatal patient population.

• Provide opportunities for medical students to interact with nurses, patients,

and families.

• Improve pediatric patient outcomes by enhancing the clinical operations of the

NICU.

At the end of this experience students will be able to:

1. Discuss risk factors, including clinical conditions and social determinants of health, that result in a NICU admission.

2. Demonstrate interpersonal skills such as working with nurses and other staff and examine the benefits of healthy interdisciplinary teamwork.

3. Practice basic skills used to comfort newborns and aid in their development.

4. Identify social and emotional stressors faced by families of infants hospitalized in the NICU.

Time Commitment:

One 2-hour cuddling shift approximately once per month, as well as additional

learning sessions and other events. Total of 10 contact hours (“cuddling”) each semester.

Training: Orientation presentation and tour of the NICU.
