# Supplementary material for: The NICU Cuddler Curriculum: A Service-Learning Curriculum for Preclinical Medical Students in the Neonatal Intensive Care Unit
Source: MedEdPORTAL. 2021 Jan 12;17:11069. doi: 10.15766/mep_2374-8265.11069 (PMC7809928; doi:10.15766/mep_2374-8265.11069)
Supplement: Supplementary file 1 — Course Description.docxParticipant Application.docxOrientation Outline.docxOrientation Presentation.pptxNeonatal Abstinence Syndrome.pptxDevelopmental Care in the NICU.pptxParent Note Cards.docxPatient Log.docxAnonymous Concerns.docxStudent Survey.docxThird- and Fourth-Year Student Survey.docxEmail to Nursing Staff.docx [file mep_2374-8265.11069-s001.zip › B. Participant Application.docx]

**NICU Cuddler Service Learning Application**

The NICU Cuddler Curriculum Program allows a select group of students to interact with patients, families, and clinicians while growing in their education and commitment to service. This program requires a committed group of students with a passion for helping the smallest patients here at **[institution]**! Aside from your required Cuddling commitment (Two hours, about once every 4-5 weeks), we also ask that you commit to the following events:
1) Attend a 2 hr orientation session
2) Participate in neonatology educational workshops taught by the NICU faculty
3) Attend the end of semester reflection

* Required

1. **Name ***

First and last name

2. **Institutional Email ***

3. **Personal email Address ***

4. **Phone number ***

5. **Briefly describe your interest in the NICU Cuddler Program ***

6. **Please describe your expectations of this program (education, commitment, and**

**responsibilities). ***

7. **Share any experience with cuddling (anything and anyone)! ***

8. **I understand this is a commitment to [insert institution] patients and families and I will make this program a**

**priority in my schedule.** *Mark only one oval.*

Agree
