# Supplementary material for: The NICU Cuddler Curriculum: A Service-Learning Curriculum for Preclinical Medical Students in the Neonatal Intensive Care Unit
Source: MedEdPORTAL. 2021 Jan 12;17:11069. doi: 10.15766/mep_2374-8265.11069 (PMC7809928; doi:10.15766/mep_2374-8265.11069)
Supplement: Supplementary file 1 — Course Description.docxParticipant Application.docxOrientation Outline.docxOrientation Presentation.pptxNeonatal Abstinence Syndrome.pptxDevelopmental Care in the NICU.pptxParent Note Cards.docxPatient Log.docxAnonymous Concerns.docxStudent Survey.docxThird- and Fourth-Year Student Survey.docxEmail to Nursing Staff.docx [file mep_2374-8265.11069-s001.zip › C. Orientation Outline.docx]

**Orientation Outline**

1. Introduce the faculty members and the student leaders.
2. 15 minute ice breaker:
   1. Everyone shares: Name, where from, what level of training, career interests, and prior experience with babies.
3. PowerPoint presentation, see Appendix J
4. Tour of NICU
   1. A tour of the NICU will be given by the faculty advisors and other staff members available.
      1. Overview of general layout of the NICU
      2. Clear demonstration of sign-in/sign-out procedure, including locations of NICU phone access, and Cuddler binder, postcards, etc.
      3. Pertinent equipment and monitors will be pointed out and explained
      4. Location of gowns and gloves, and instructions regarding formal contact precautions and general NICU infection prevention standards will be discussed.
   2. After the initial orientation to the floor, the group should be broken up into smaller groups of about 5 students.
   3. The staff members should take their group into a room with a high-acuity patient to demonstrate the range of acuity in the NICU, as they will be cuddling lower acuity patients.
   4. Then they should be taken into a room with a patient that is appropriate for cuddling, who has been already determined after a discussion with the staff and ideally the family, if appropriate.
      1. The staff member should then demonstrate how to properly cuddle a baby, including introducing themselves to the nursing staff (role play) and modeling transitioning the baby to the holder’s arms. One or two students should don a gown and gloves and practice the interaction.
         1. Important points:
            1. Access to a call or emergency button
            2. Comfort of holder: Arm supports
            3. Suggested activities with baby: Quiet talking, singing, reading
            4. Not allowed: Food, drink, or handling cell phones while cuddling
            5. Reading baby cues
            6. Normalize the stress from holding a crying baby

Make it clear that they can put the baby back if the stress is too much.

- 1. There should be adequate time for any questions the students may have.

1. Collect all the contact information for the students so that access to the google calendar (or however you choose to schedule the students) can be made available so they know when their shifts will be.
