# Supplementary material for: The NICU Cuddler Curriculum: A Service-Learning Curriculum for Preclinical Medical Students in the Neonatal Intensive Care Unit
Source: MedEdPORTAL. 2021 Jan 12;17:11069. doi: 10.15766/mep_2374-8265.11069 (PMC7809928; doi:10.15766/mep_2374-8265.11069)

## Slide 1
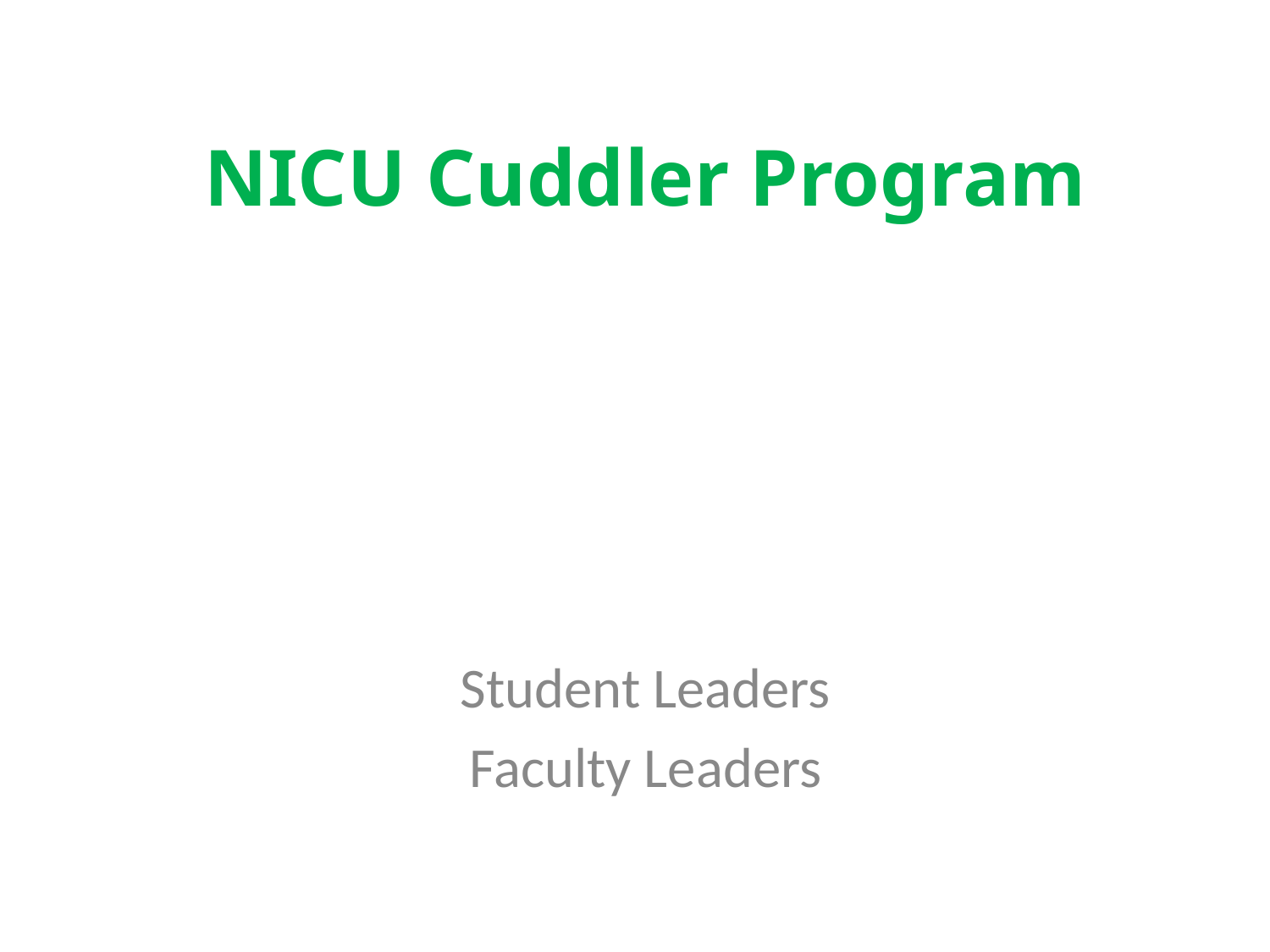

# NICU Cuddler Program
Student Leaders
Faculty Leaders

## Slide 2
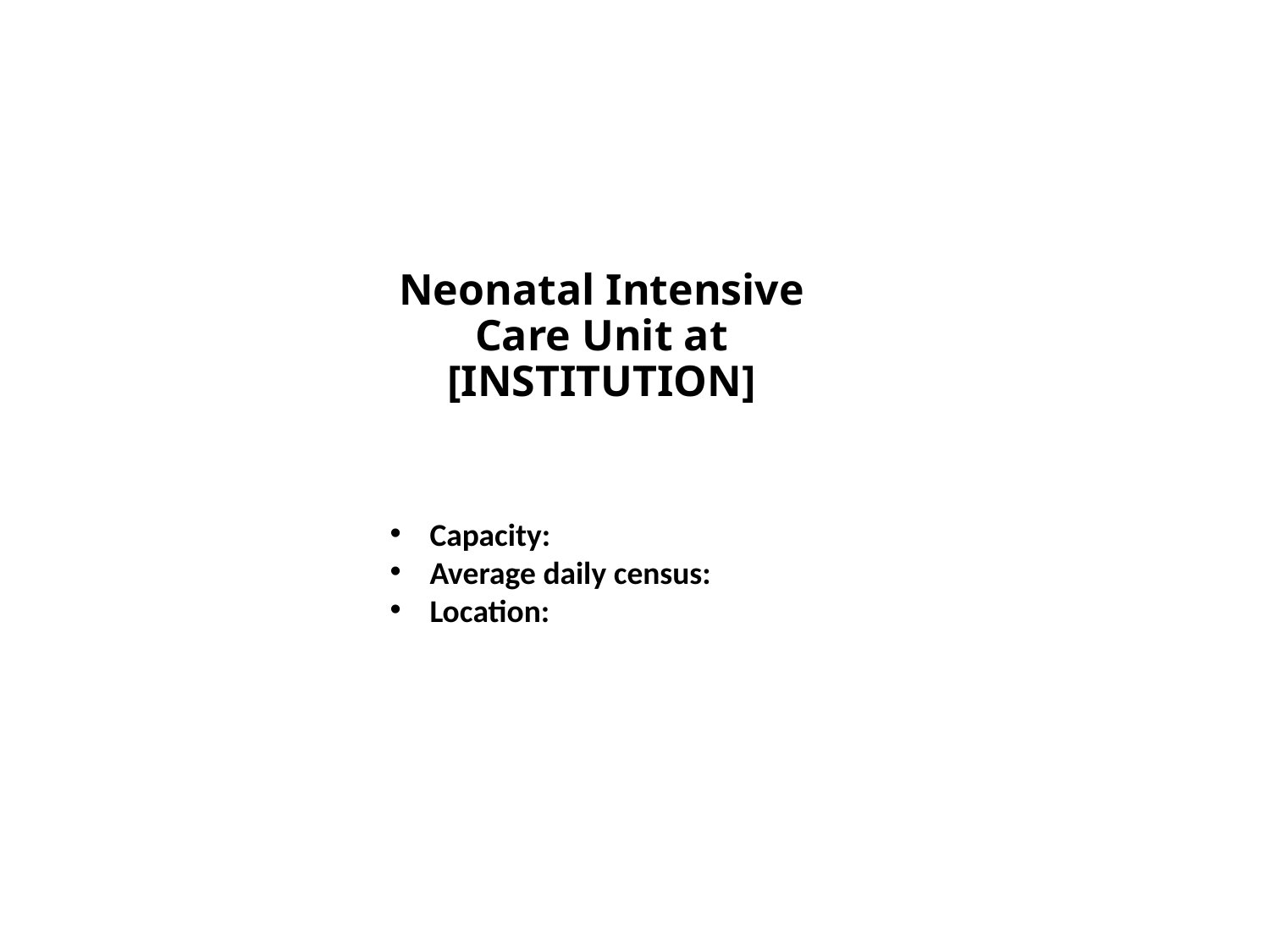

# Neonatal Intensive Care Unit at [INSTITUTION]
Capacity:
Average daily census:
Location:

## Slide 3
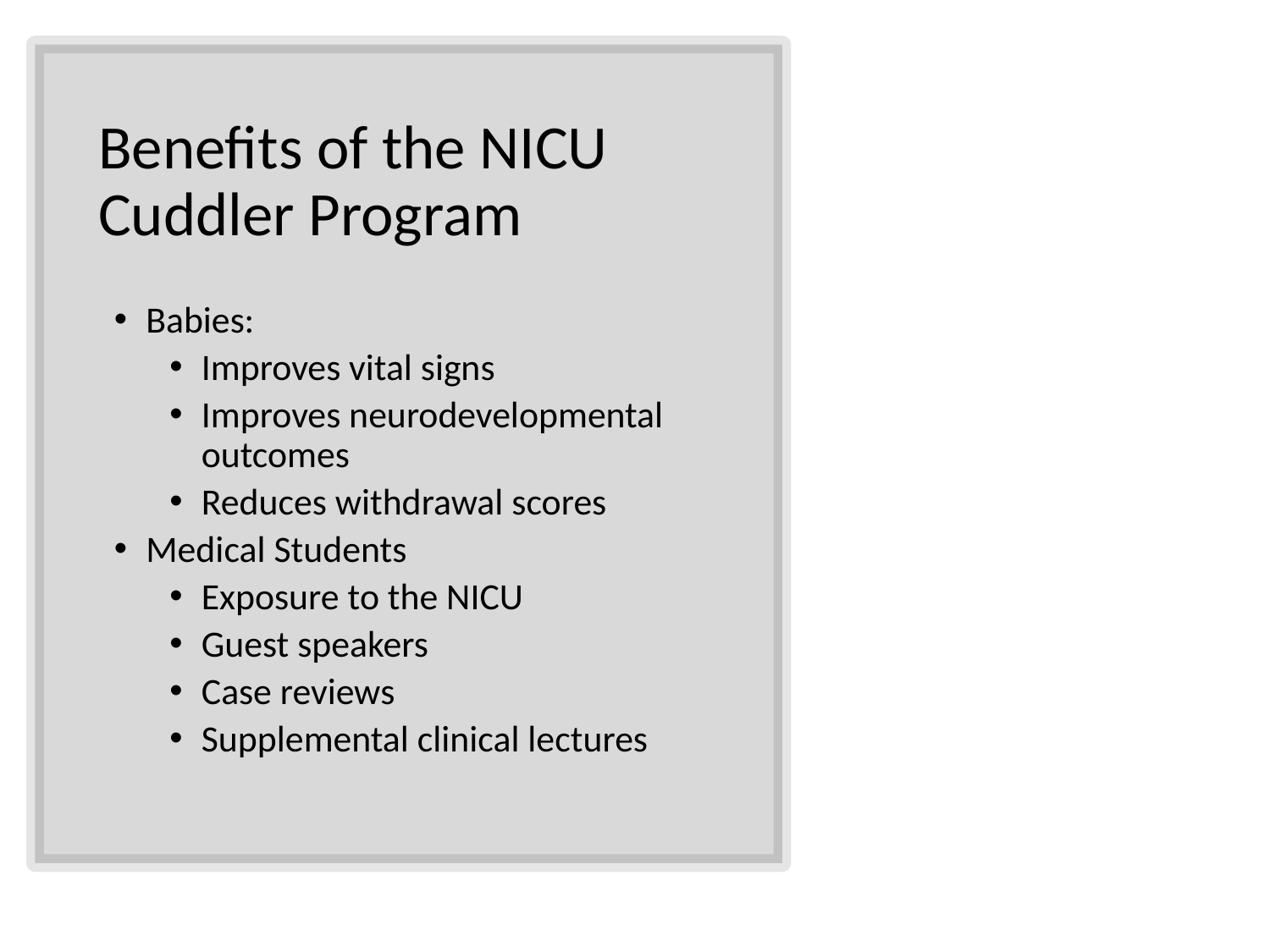

# Benefits of the NICU Cuddler Program
Babies:
Improves vital signs
Improves neurodevelopmental outcomes
Reduces withdrawal scores
Medical Students
Exposure to the NICU
Guest speakers
Case reviews
Supplemental clinical lectures

## Slide 4
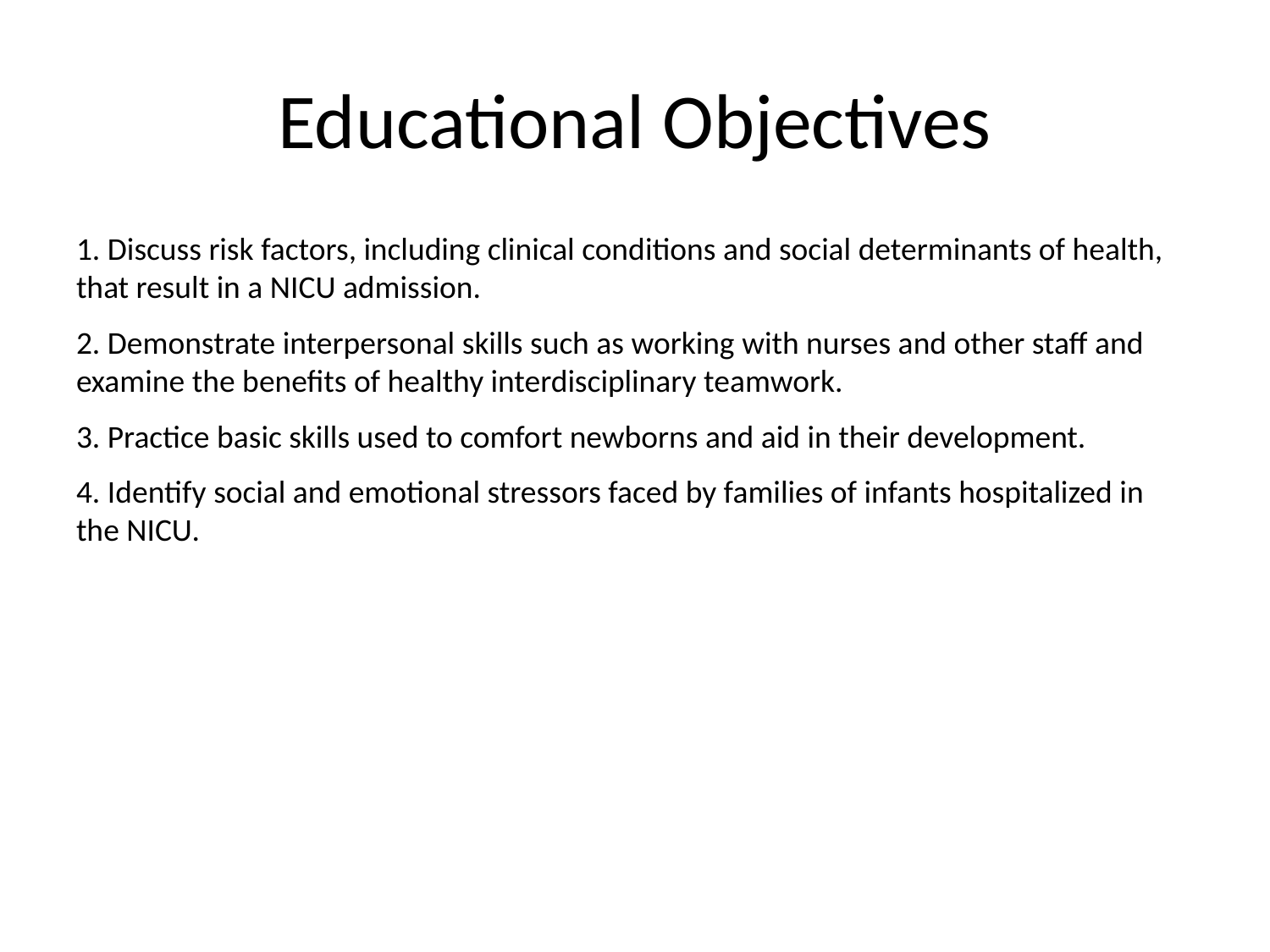

# Educational Objectives
1. Discuss risk factors, including clinical conditions and social determinants of health, that result in a NICU admission.
2. Demonstrate interpersonal skills such as working with nurses and other staff and examine the benefits of healthy interdisciplinary teamwork.
3. Practice basic skills used to comfort newborns and aid in their development.
4. Identify social and emotional stressors faced by families of infants hospitalized in the NICU.

## Slide 5
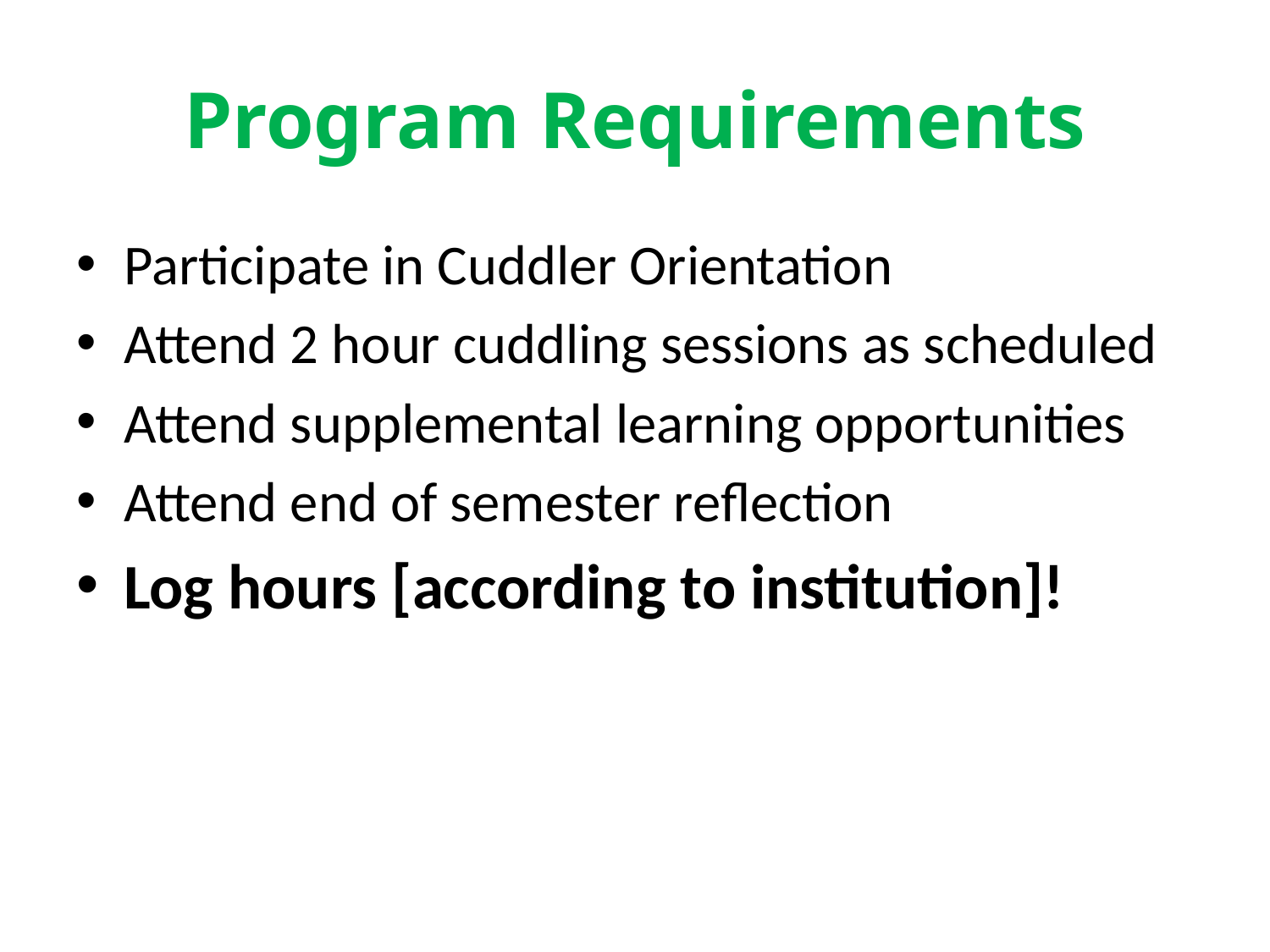

# Program Requirements
Participate in Cuddler Orientation
Attend 2 hour cuddling sessions as scheduled
Attend supplemental learning opportunities
Attend end of semester reflection
Log hours [according to institution]!

## Slide 6
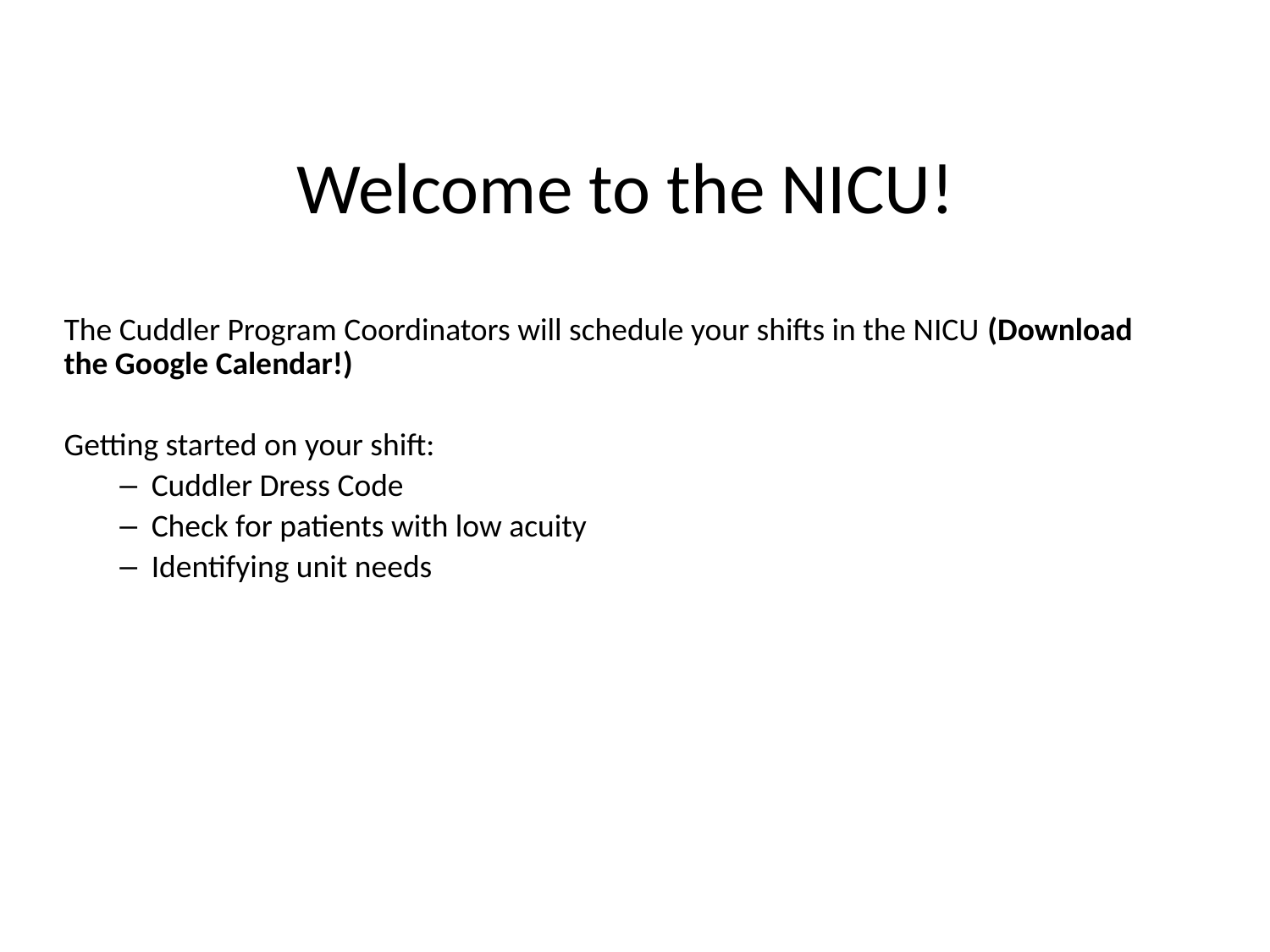

# Welcome to the NICU!
The Cuddler Program Coordinators will schedule your shifts in the NICU (Download the Google Calendar!)
Getting started on your shift:
Cuddler Dress Code
Check for patients with low acuity
Identifying unit needs

## Slide 7
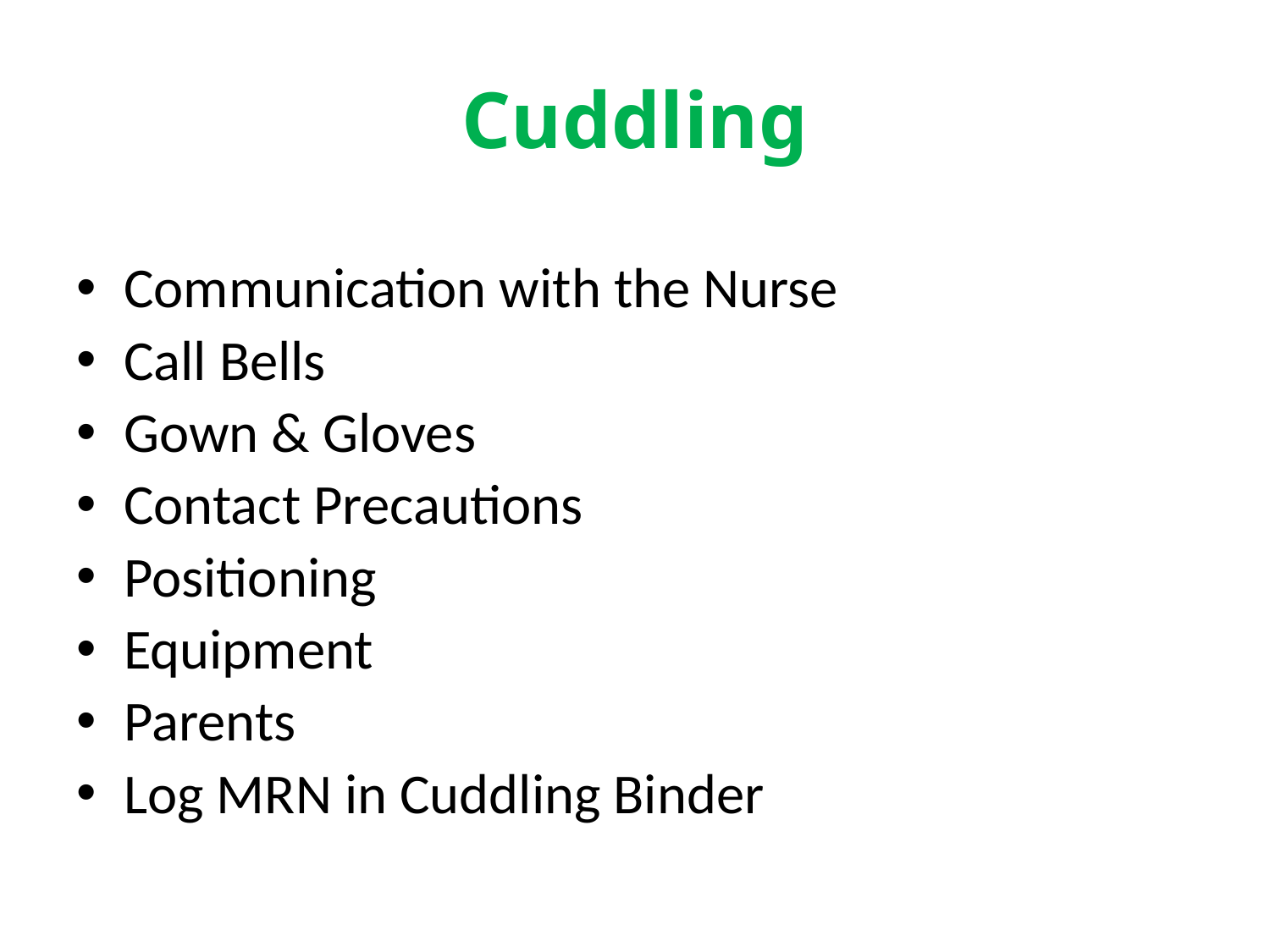

# Cuddling
Communication with the Nurse
Call Bells
Gown & Gloves
Contact Precautions
Positioning
Equipment
Parents
Log MRN in Cuddling Binder

## Slide 8
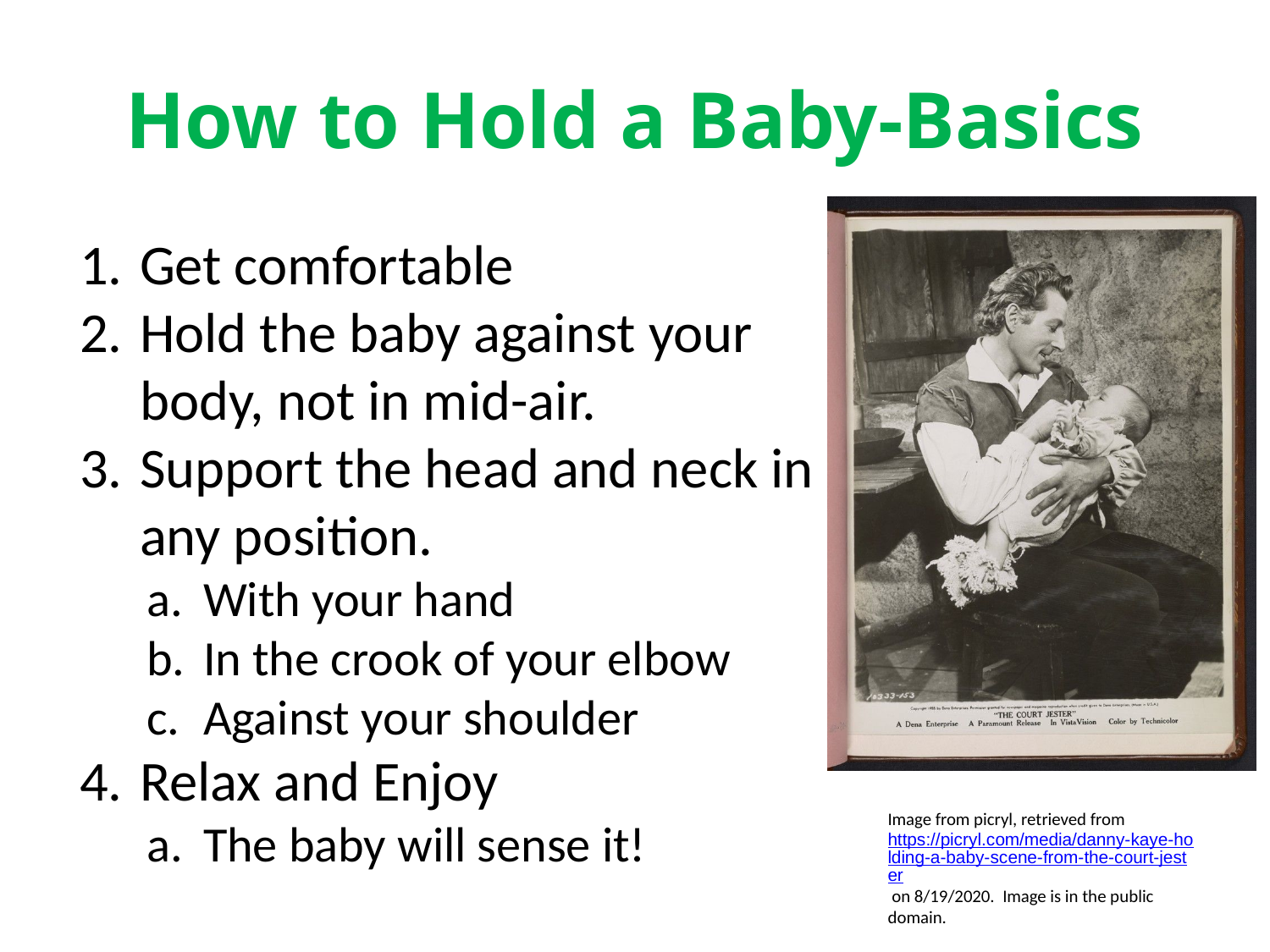

# How to Hold a Baby-Basics
Get comfortable
Hold the baby against your body, not in mid-air.
Support the head and neck in any position.
With your hand
In the crook of your elbow
Against your shoulder
Relax and Enjoy
The baby will sense it!
Image from picryl, retrieved from https://picryl.com/media/danny-kaye-holding-a-baby-scene-from-the-court-jester on 8/19/2020. Image is in the public domain.

## Slide 9
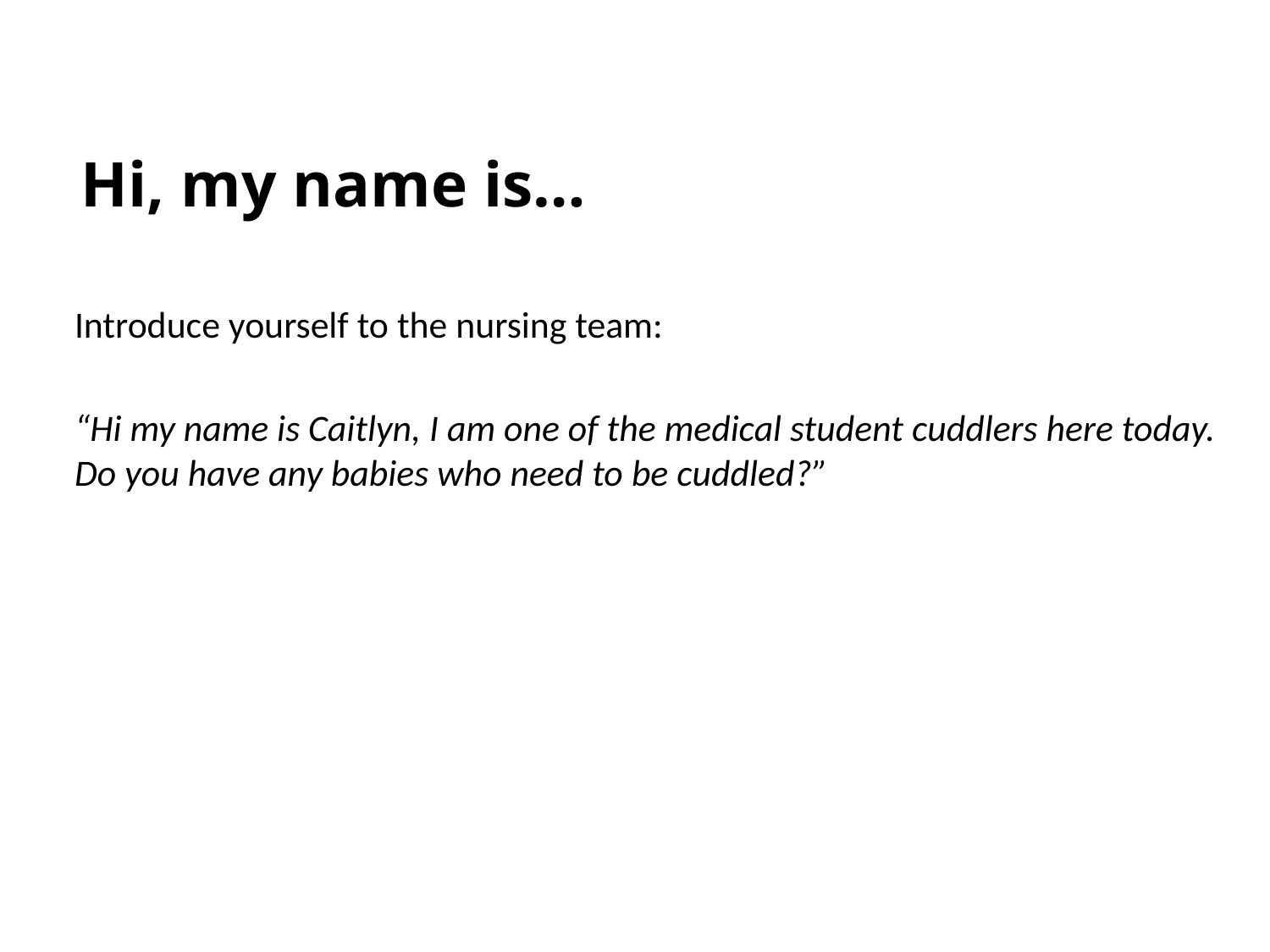

# Hi, my name is…
Introduce yourself to the nursing team:
“Hi my name is Caitlyn, I am one of the medical student cuddlers here today. Do you have any babies who need to be cuddled?”

## Slide 10
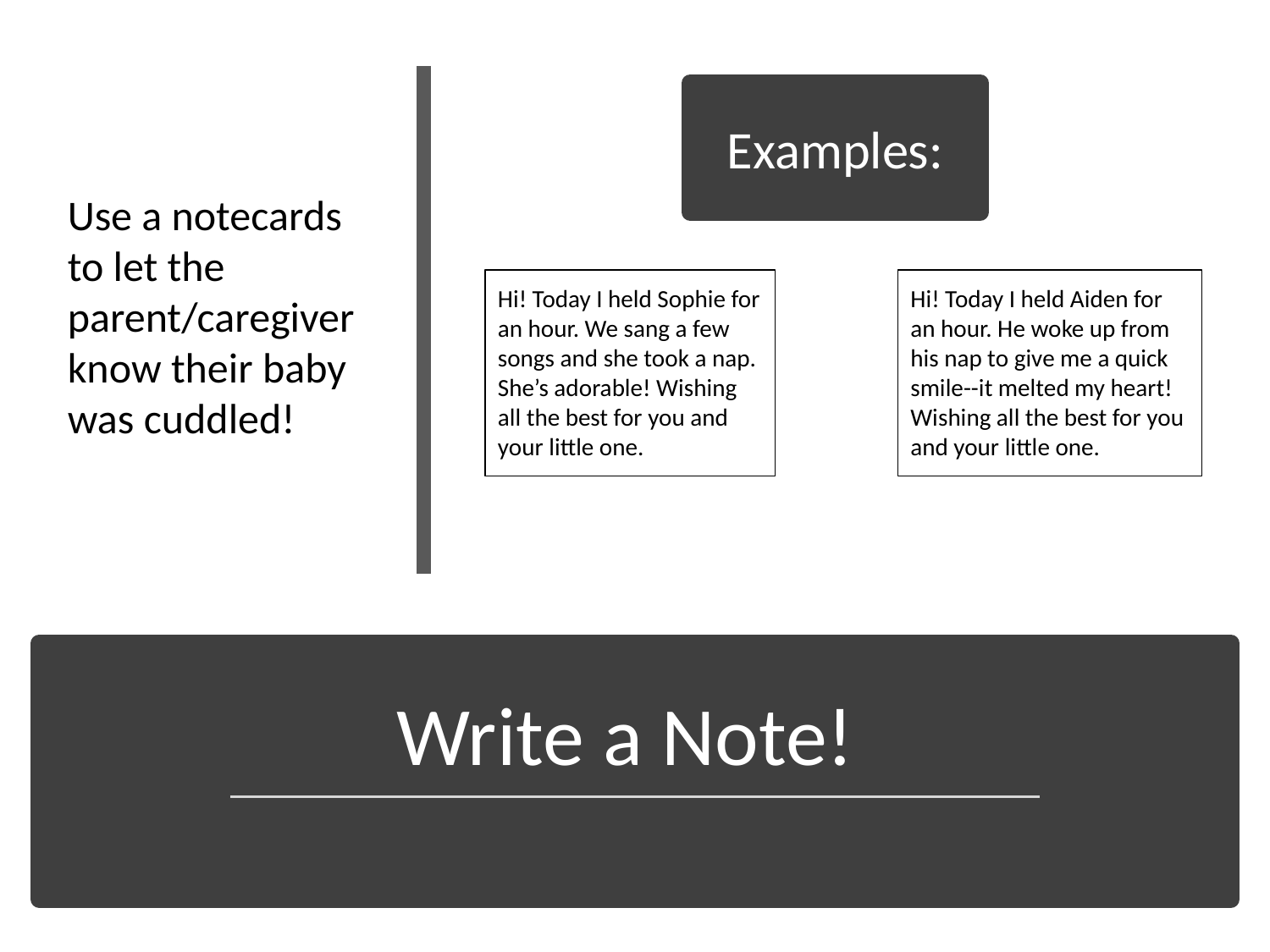

Examples:
Use a notecards to let the parent/caregiver know their baby was cuddled!
Hi! Today I held Sophie for an hour. We sang a few songs and she took a nap. She’s adorable! Wishing all the best for you and your little one.
Hi! Today I held Aiden for an hour. He woke up from his nap to give me a quick smile--it melted my heart! Wishing all the best for you and your little one.
# Write a Note!

## Slide 11
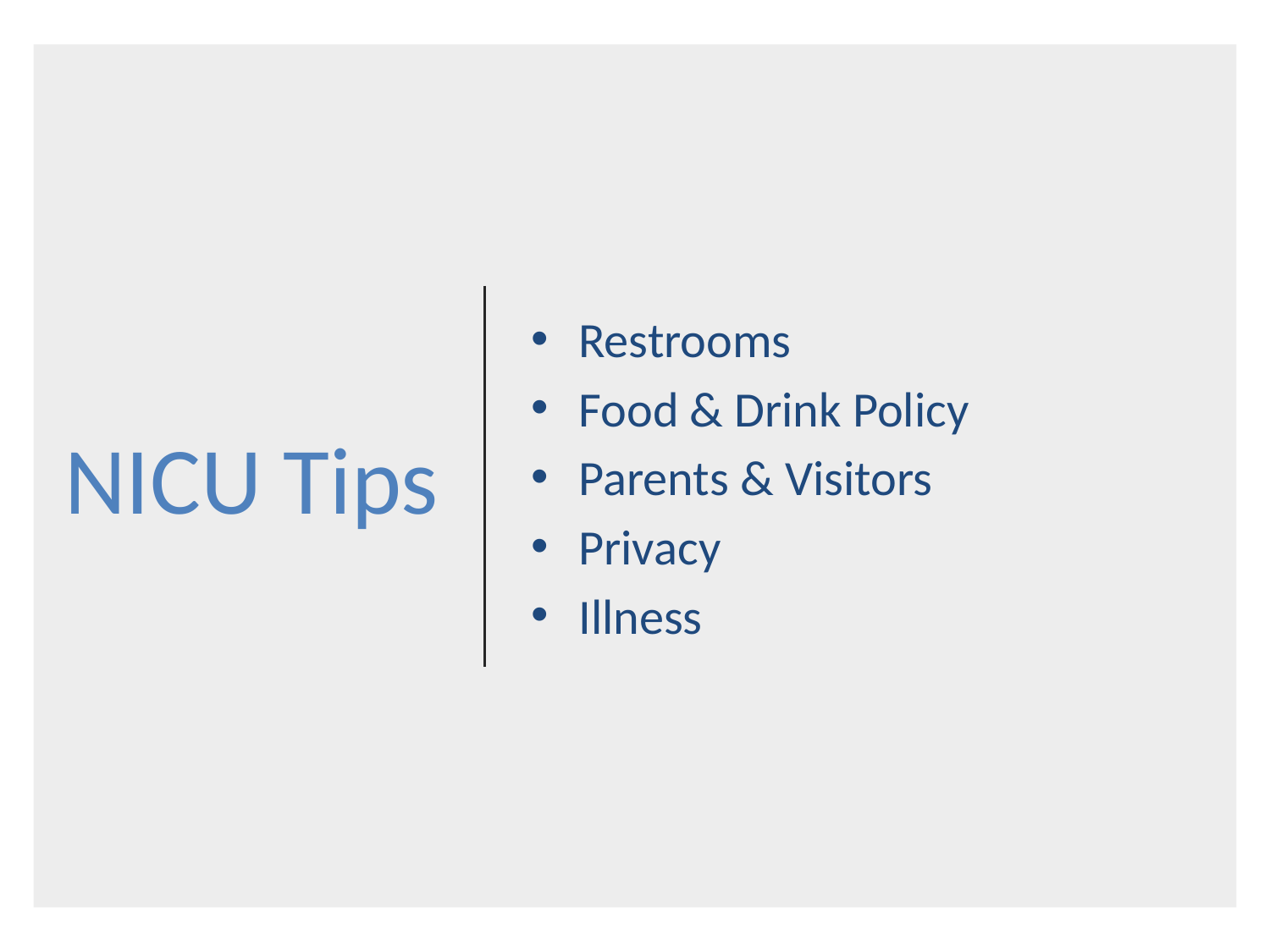

# NICU Tips
Restrooms
Food & Drink Policy
Parents & Visitors
Privacy
Illness

## Slide 12
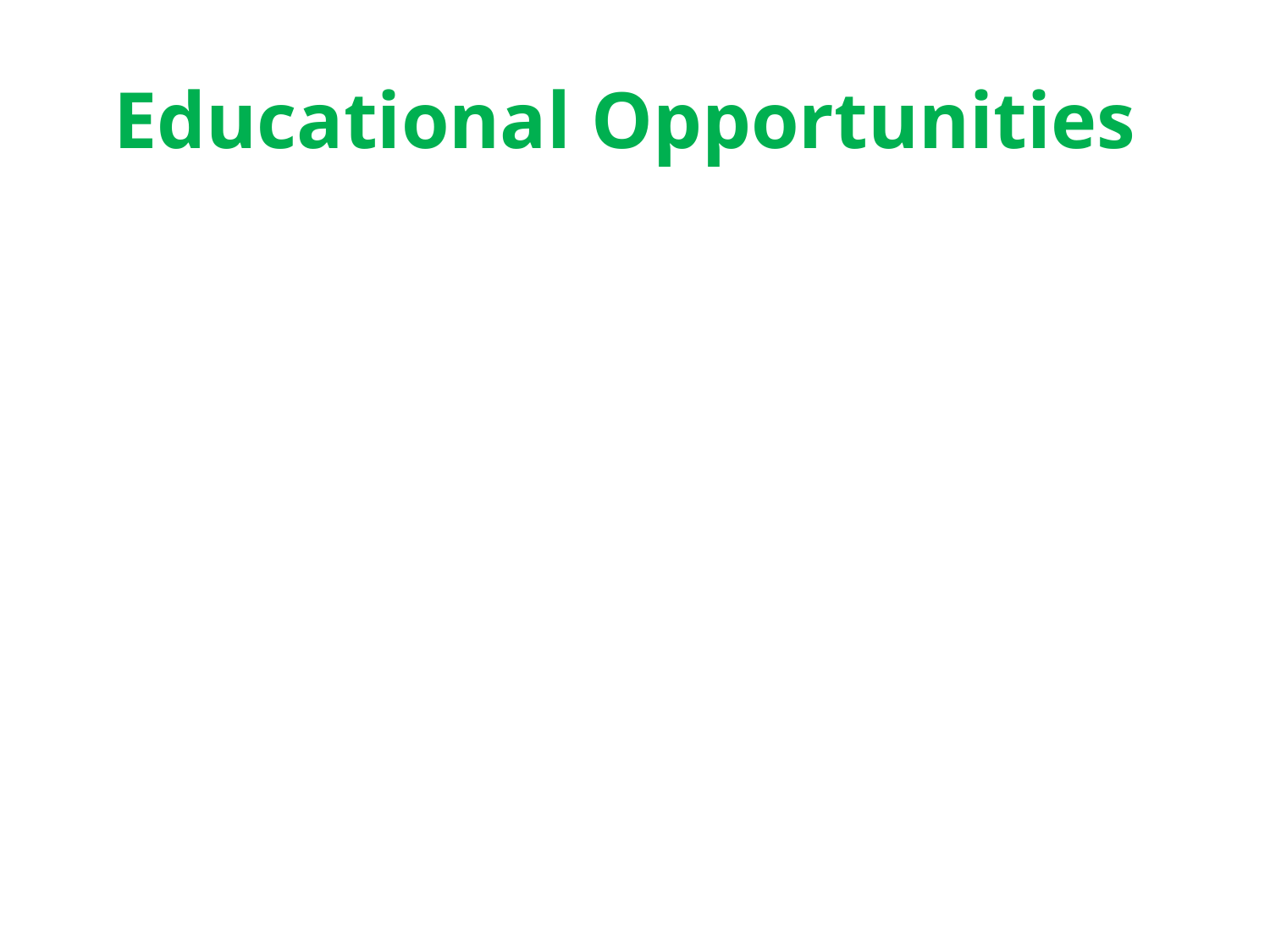

# Educational Opportunities

## Slide 13
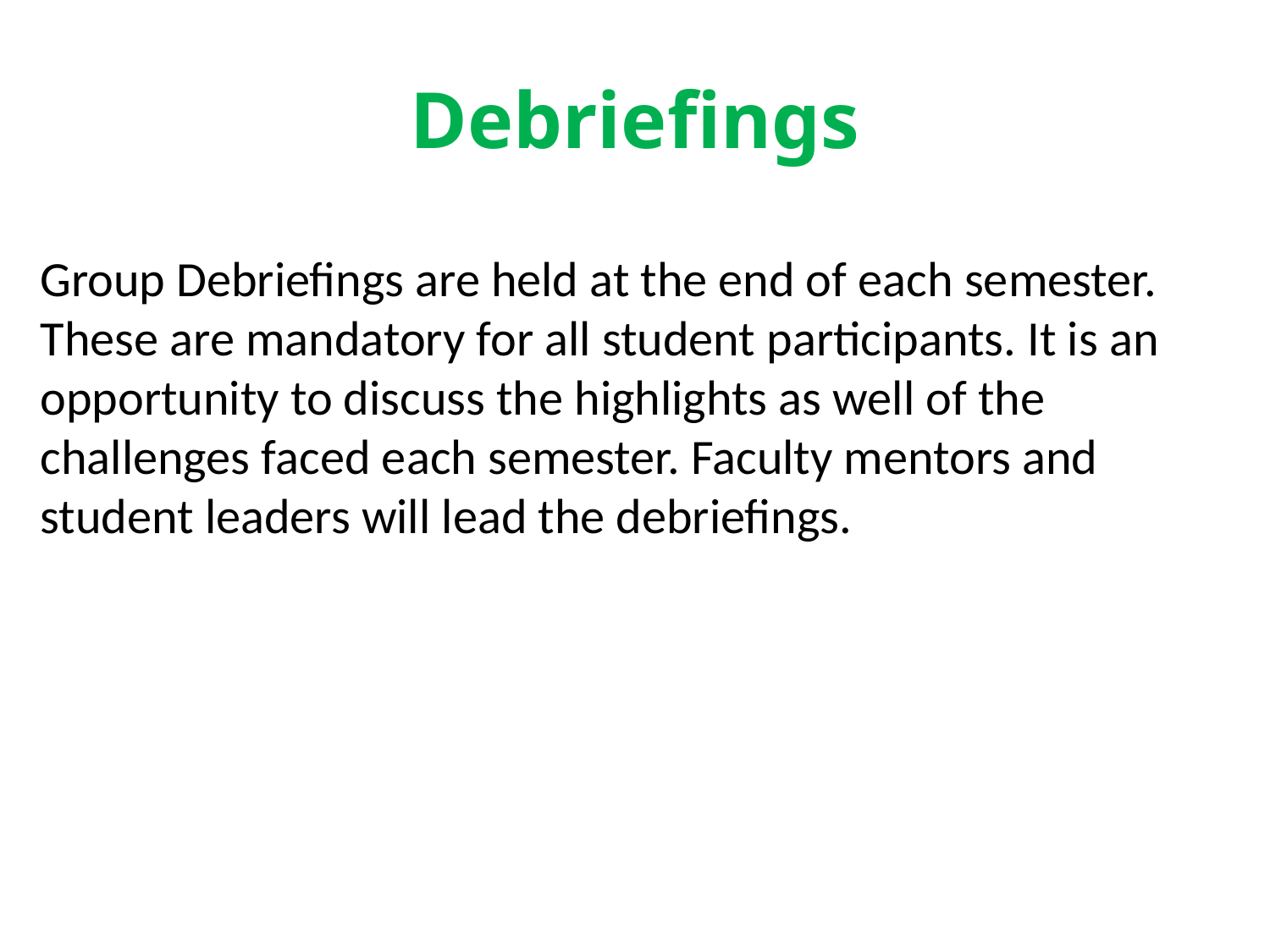

# Debriefings
Group Debriefings are held at the end of each semester. These are mandatory for all student participants. It is an opportunity to discuss the highlights as well of the challenges faced each semester. Faculty mentors and student leaders will lead the debriefings.

## Slide 14
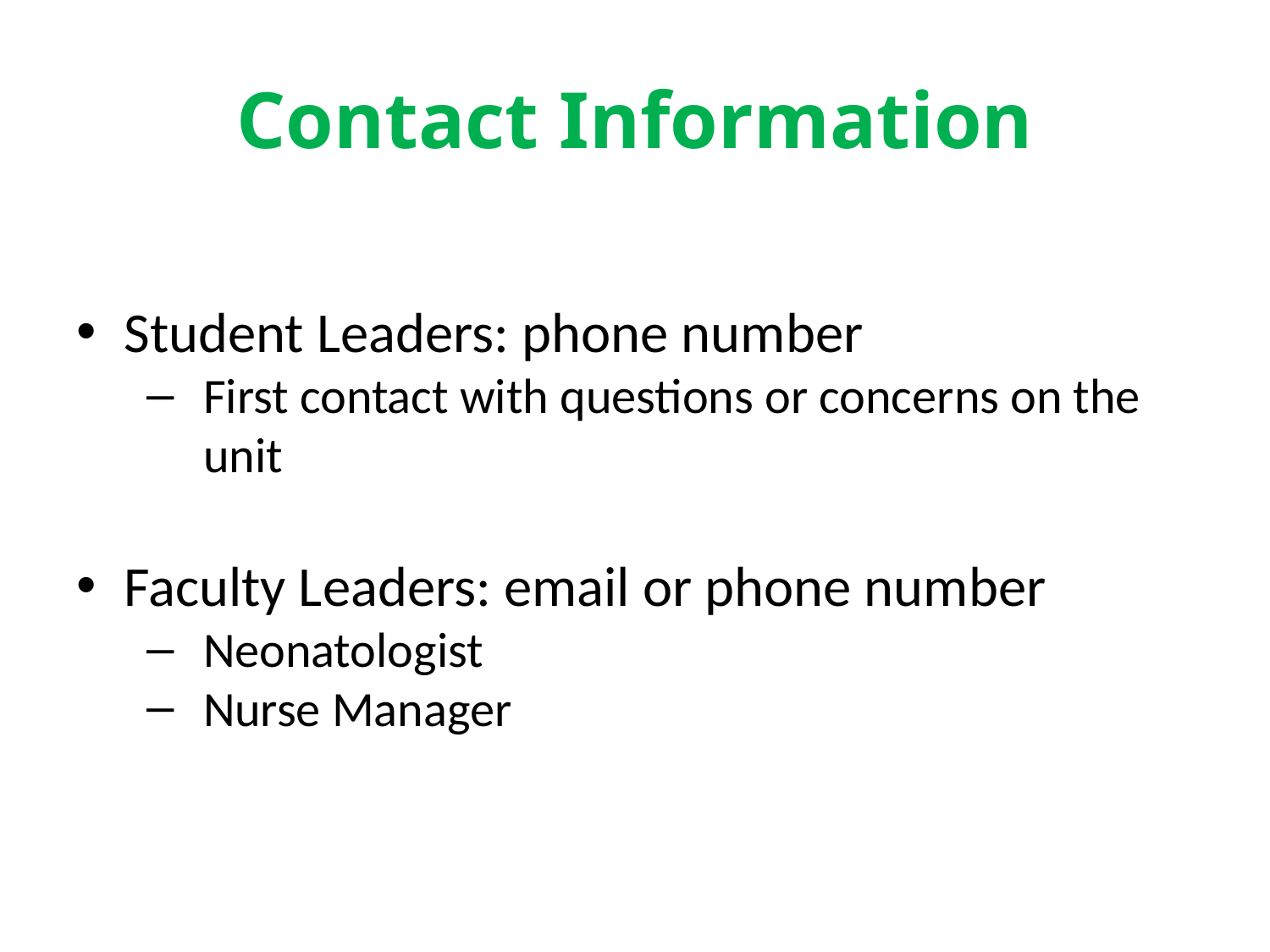

# Contact Information
Student Leaders: phone number
First contact with questions or concerns on the unit
Faculty Leaders: email or phone number
Neonatologist
Nurse Manager

## Slide 15
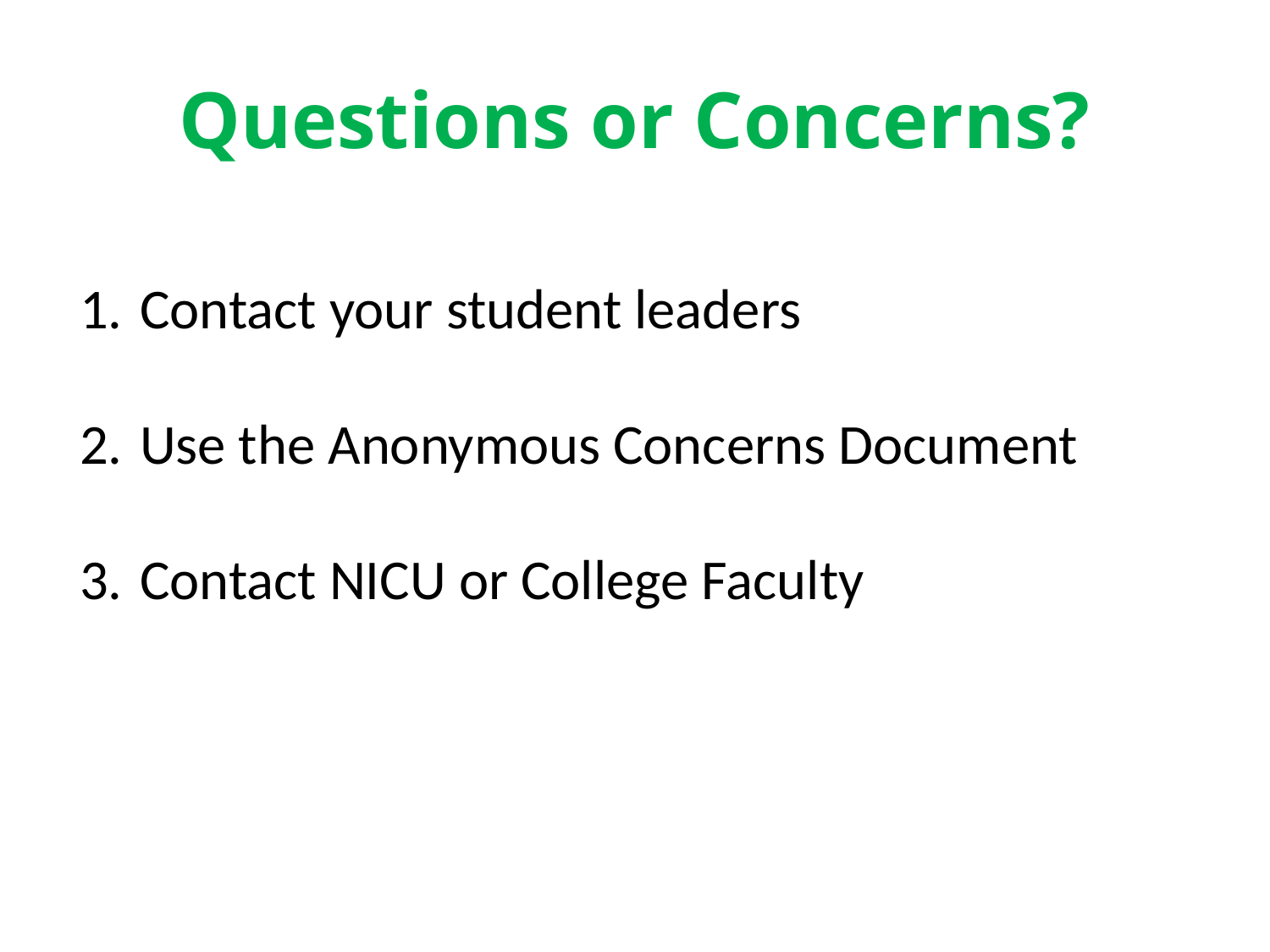

# Questions or Concerns?
Contact your student leaders
Use the Anonymous Concerns Document
Contact NICU or College Faculty

## Slide 16
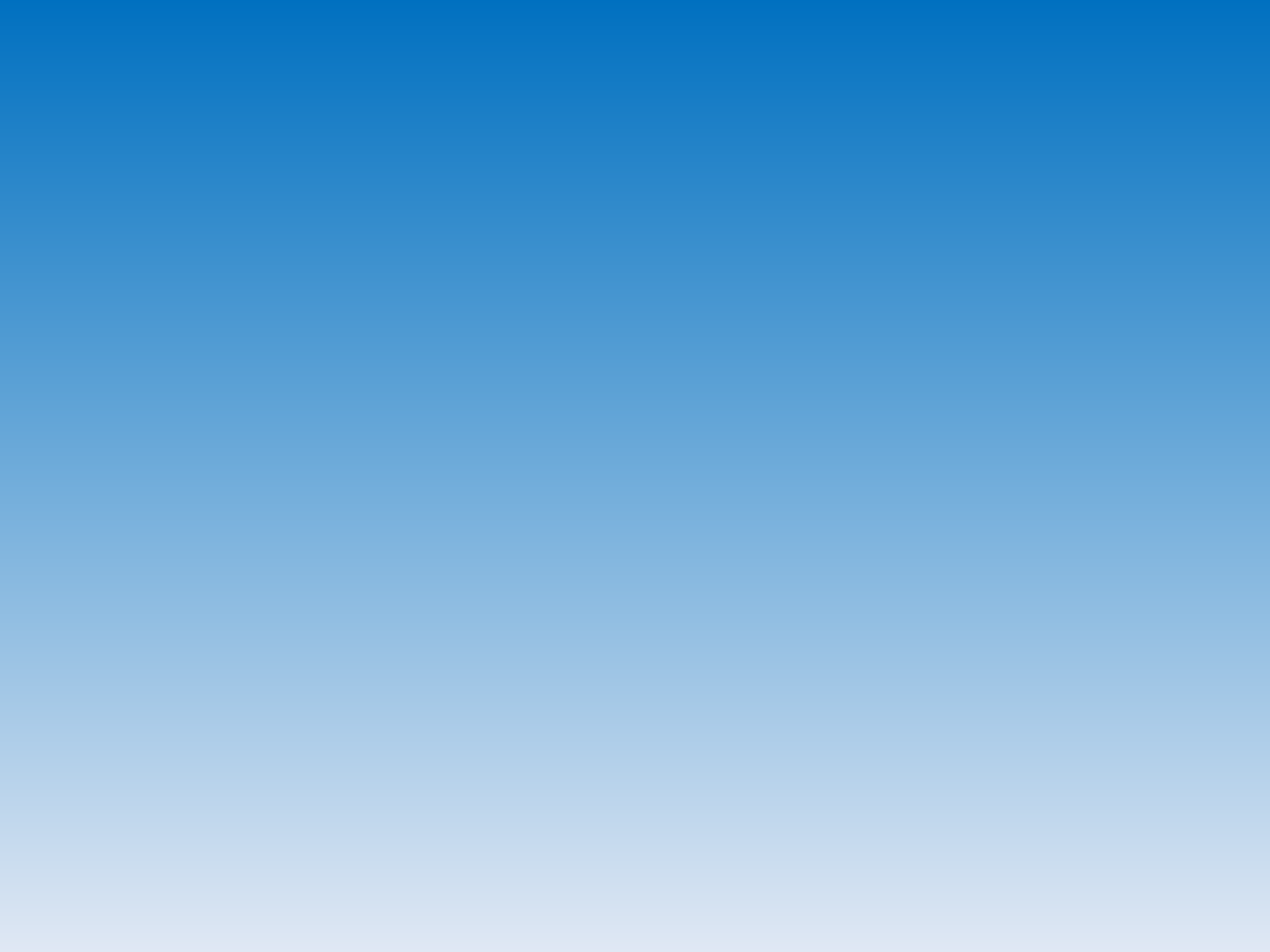

Supplement: Supplementary file 1 — Course Description.docxParticipant Application.docxOrientation Outline.docxOrientation Presentation.pptxNeonatal Abstinence Syndrome.pptxDevelopmental Care in the NICU.pptxParent Note Cards.docxPatient Log.docxAnonymous Concerns.docxStudent Survey.docxThird- and Fourth-Year Student Survey.docxEmail to Nursing Staff.docx [file mep_2374-8265.11069-s001.zip › D. Orientation Presentation.pptx]
