# Supplementary material for: The NICU Cuddler Curriculum: A Service-Learning Curriculum for Preclinical Medical Students in the Neonatal Intensive Care Unit
Source: MedEdPORTAL. 2021 Jan 12;17:11069. doi: 10.15766/mep_2374-8265.11069 (PMC7809928; doi:10.15766/mep_2374-8265.11069)
Supplement: Supplementary file 1 — Course Description.docxParticipant Application.docxOrientation Outline.docxOrientation Presentation.pptxNeonatal Abstinence Syndrome.pptxDevelopmental Care in the NICU.pptxParent Note Cards.docxPatient Log.docxAnonymous Concerns.docxStudent Survey.docxThird- and Fourth-Year Student Survey.docxEmail to Nursing Staff.docx [file mep_2374-8265.11069-s001.zip › E. Neonatal Abstinence Syndrome.pptx]

## Slide 1
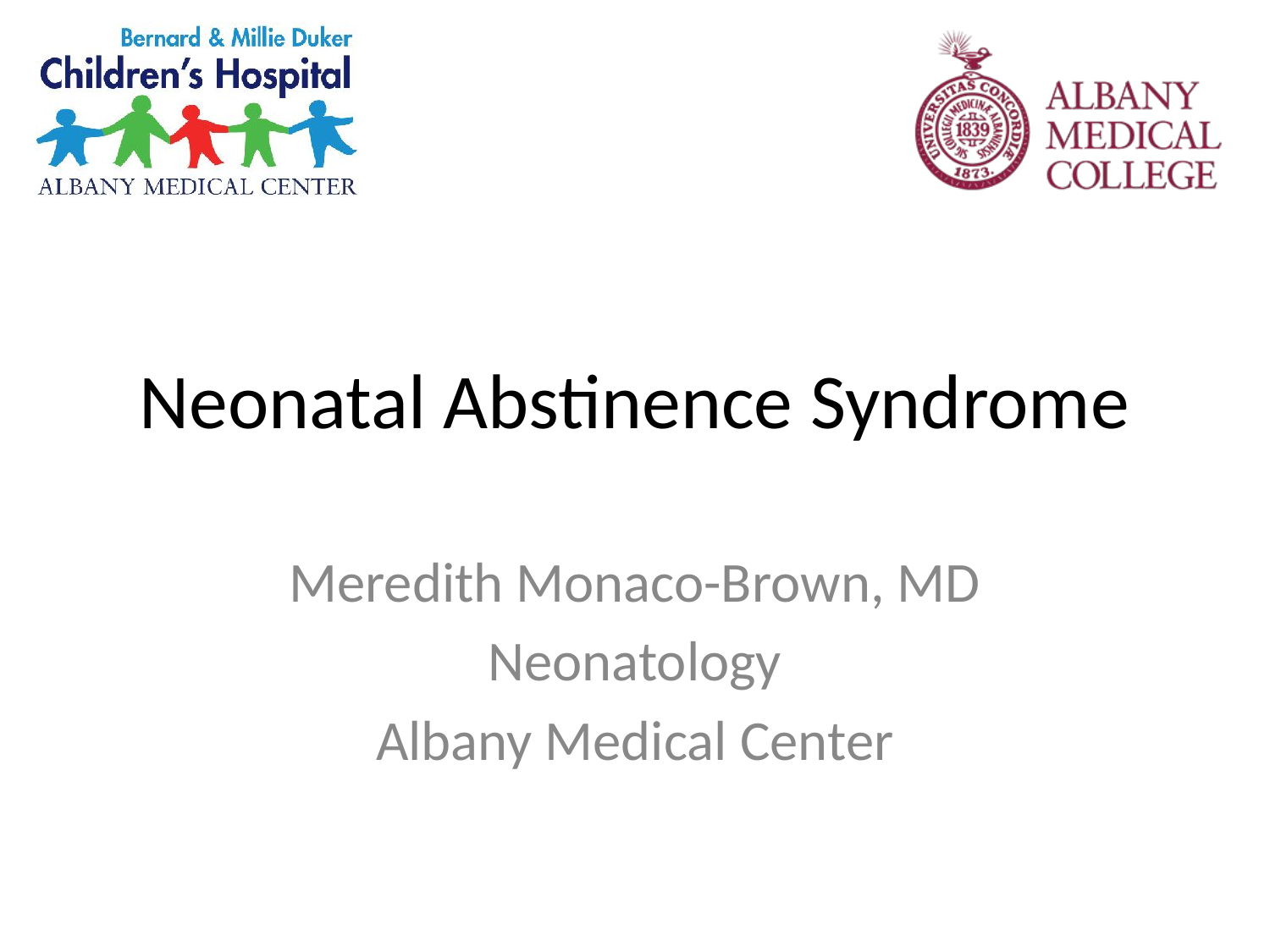

# Neonatal Abstinence Syndrome
Meredith Monaco-Brown, MD
Neonatology
Albany Medical Center

## Slide 2
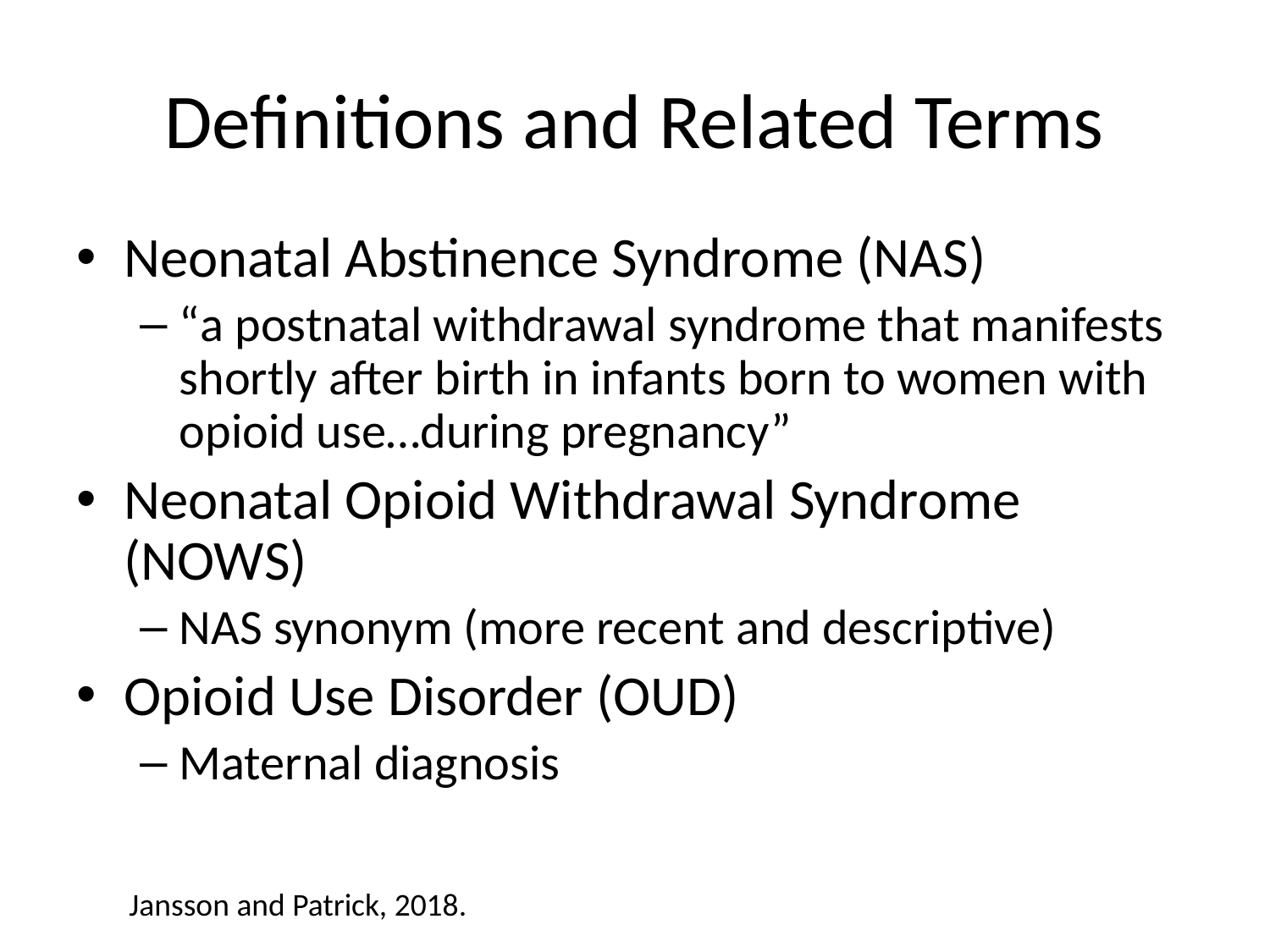

# Definitions and Related Terms
Neonatal Abstinence Syndrome (NAS)
“a postnatal withdrawal syndrome that manifests shortly after birth in infants born to women with opioid use…during pregnancy”
Neonatal Opioid Withdrawal Syndrome (NOWS)
NAS synonym (more recent and descriptive)
Opioid Use Disorder (OUD)
Maternal diagnosis
Jansson and Patrick, 2018.

## Slide 3
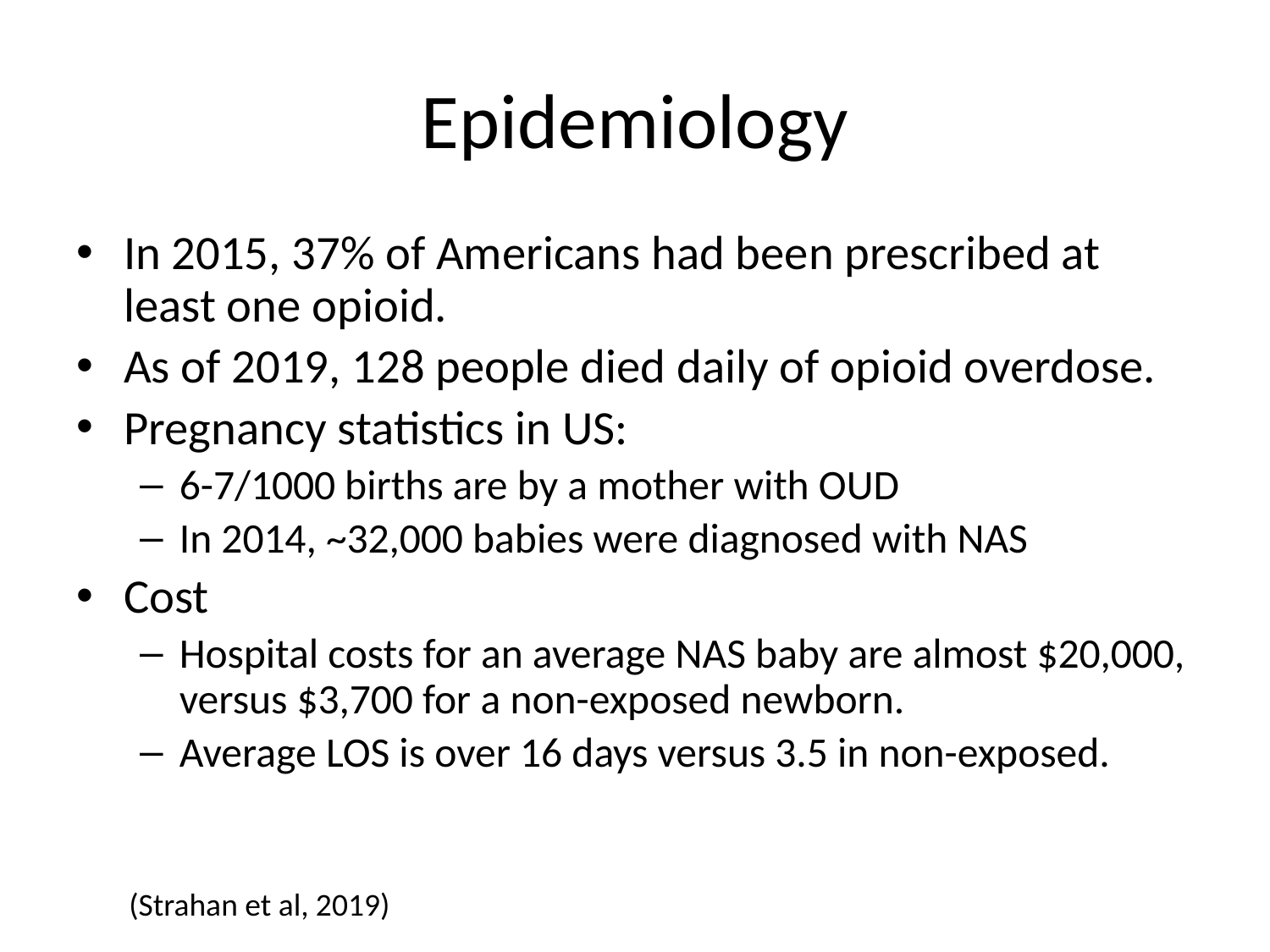

# Epidemiology
In 2015, 37% of Americans had been prescribed at least one opioid.
As of 2019, 128 people died daily of opioid overdose.
Pregnancy statistics in US:
6-7/1000 births are by a mother with OUD
In 2014, ~32,000 babies were diagnosed with NAS
Cost
Hospital costs for an average NAS baby are almost $20,000, versus $3,700 for a non-exposed newborn.
Average LOS is over 16 days versus 3.5 in non-exposed.
(Strahan et al, 2019)

## Slide 4
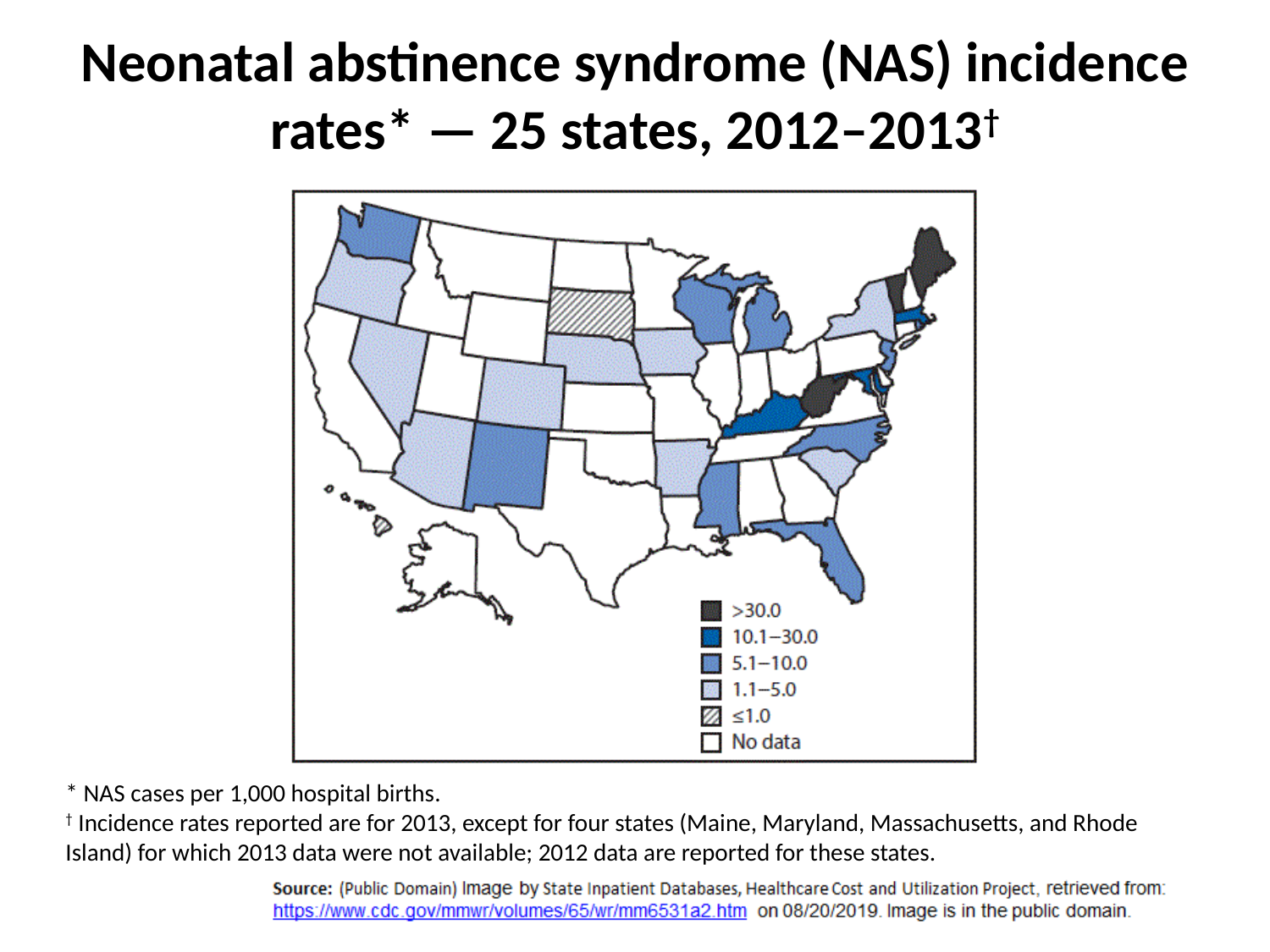

# Neonatal abstinence syndrome (NAS) incidence rates* — 25 states, 2012–2013†
* NAS cases per 1,000 hospital births.
† Incidence rates reported are for 2013, except for four states (Maine, Maryland, Massachusetts, and Rhode Island) for which 2013 data were not available; 2012 data are reported for these states.

## Slide 5
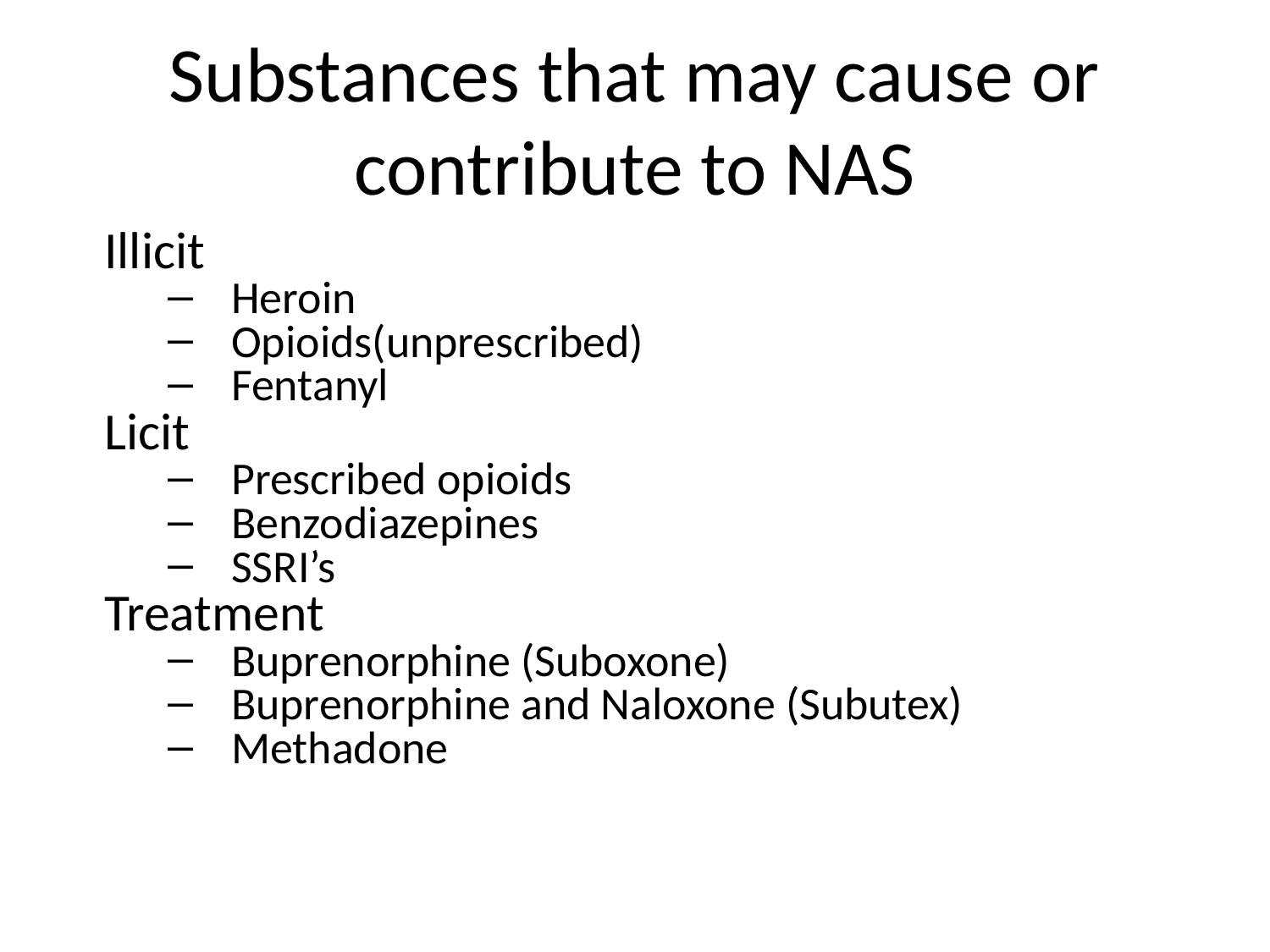

# Substances that may cause or contribute to NAS
Illicit
Heroin
Opioids(unprescribed)
Fentanyl
Licit
Prescribed opioids
Benzodiazepines
SSRI’s
Treatment
Buprenorphine (Suboxone)
Buprenorphine and Naloxone (Subutex)
Methadone

## Slide 6
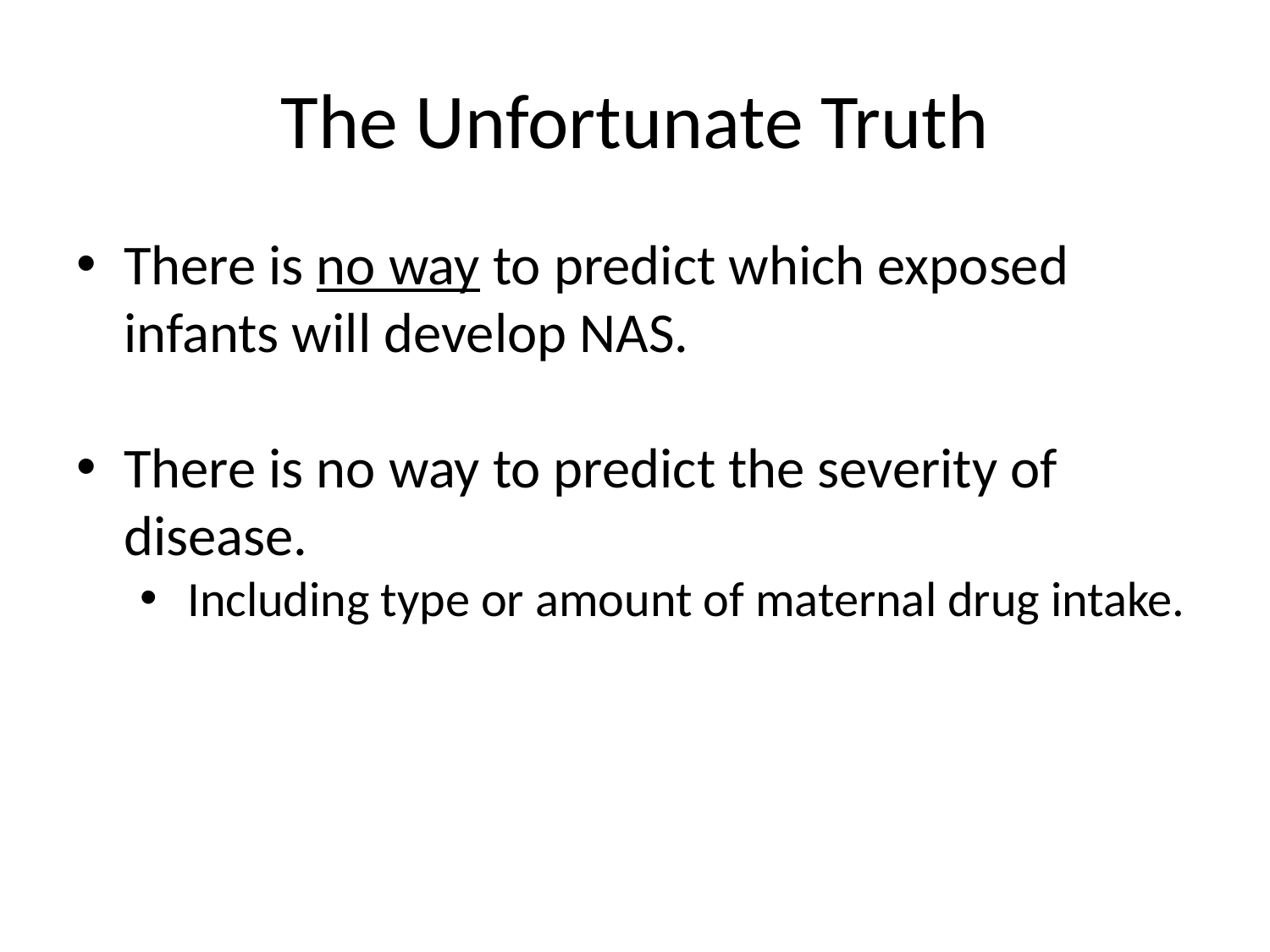

# The Unfortunate Truth
There is no way to predict which exposed infants will develop NAS.
There is no way to predict the severity of disease.
Including type or amount of maternal drug intake.

## Slide 7
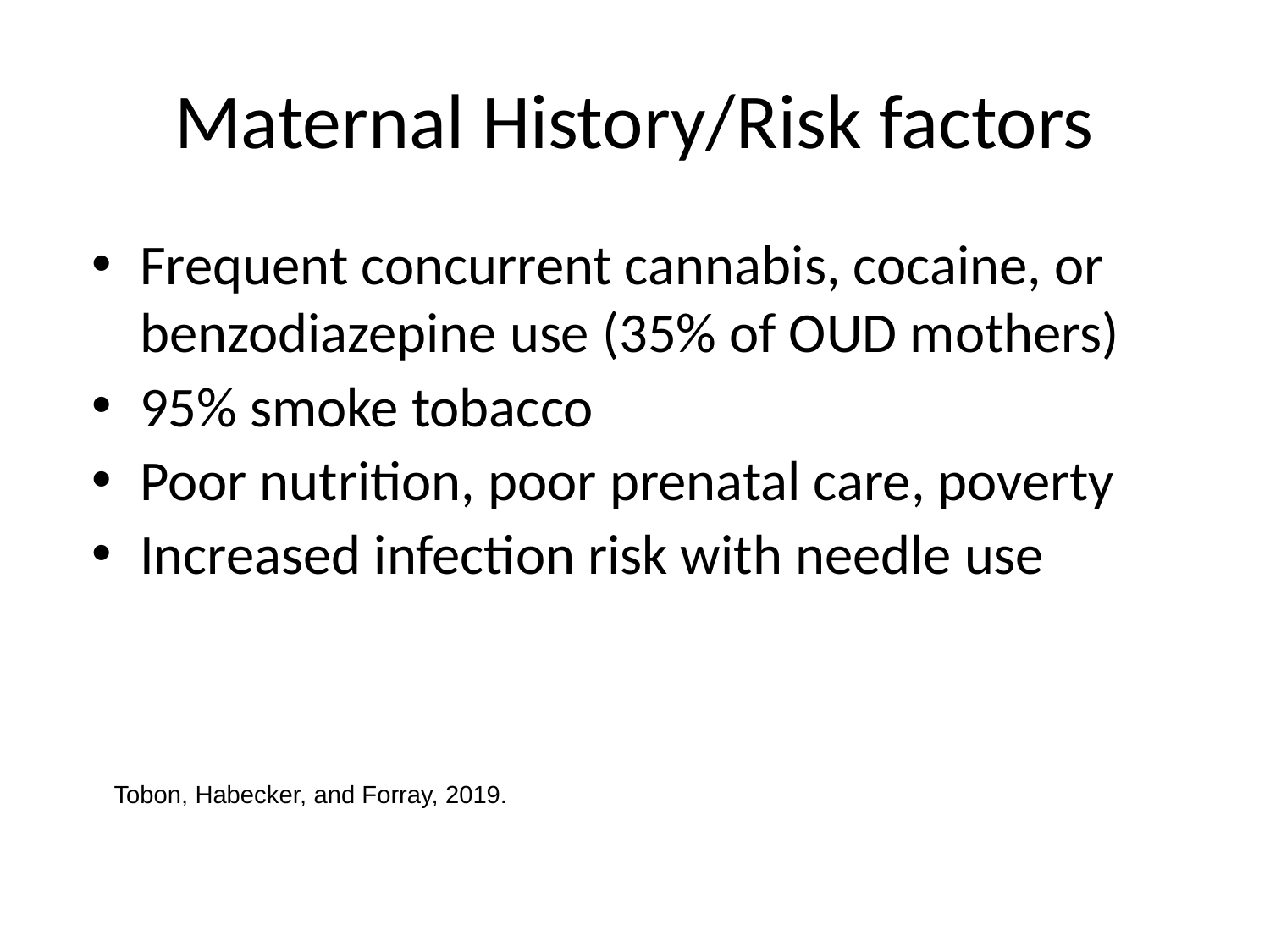

# Maternal History/Risk factors
Frequent concurrent cannabis, cocaine, or benzodiazepine use (35% of OUD mothers)
95% smoke tobacco
Poor nutrition, poor prenatal care, poverty
Increased infection risk with needle use
Tobon, Habecker, and Forray, 2019.

## Slide 8
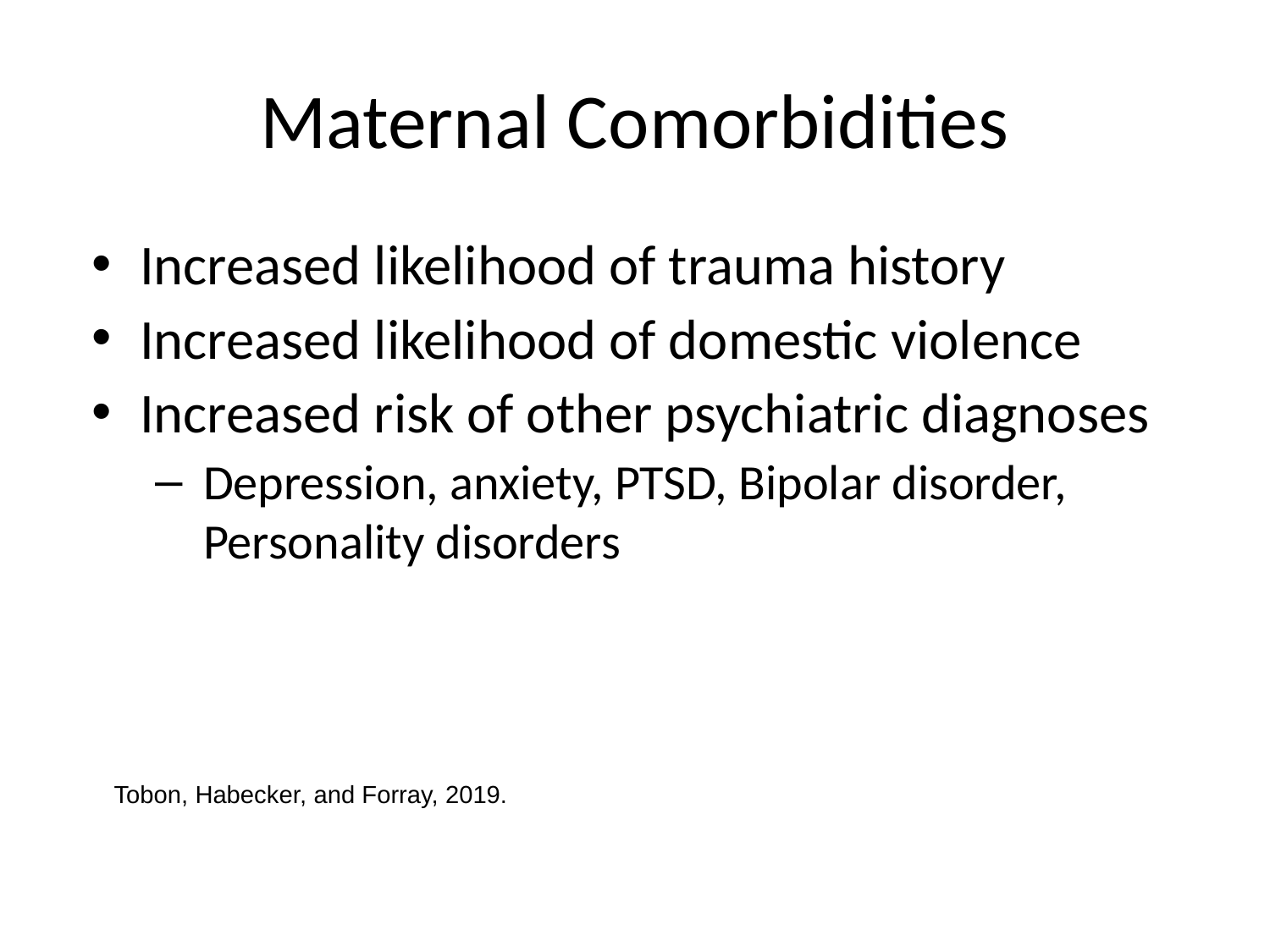

# Maternal Comorbidities
Increased likelihood of trauma history
Increased likelihood of domestic violence
Increased risk of other psychiatric diagnoses
Depression, anxiety, PTSD, Bipolar disorder, Personality disorders
Tobon, Habecker, and Forray, 2019.

## Slide 9
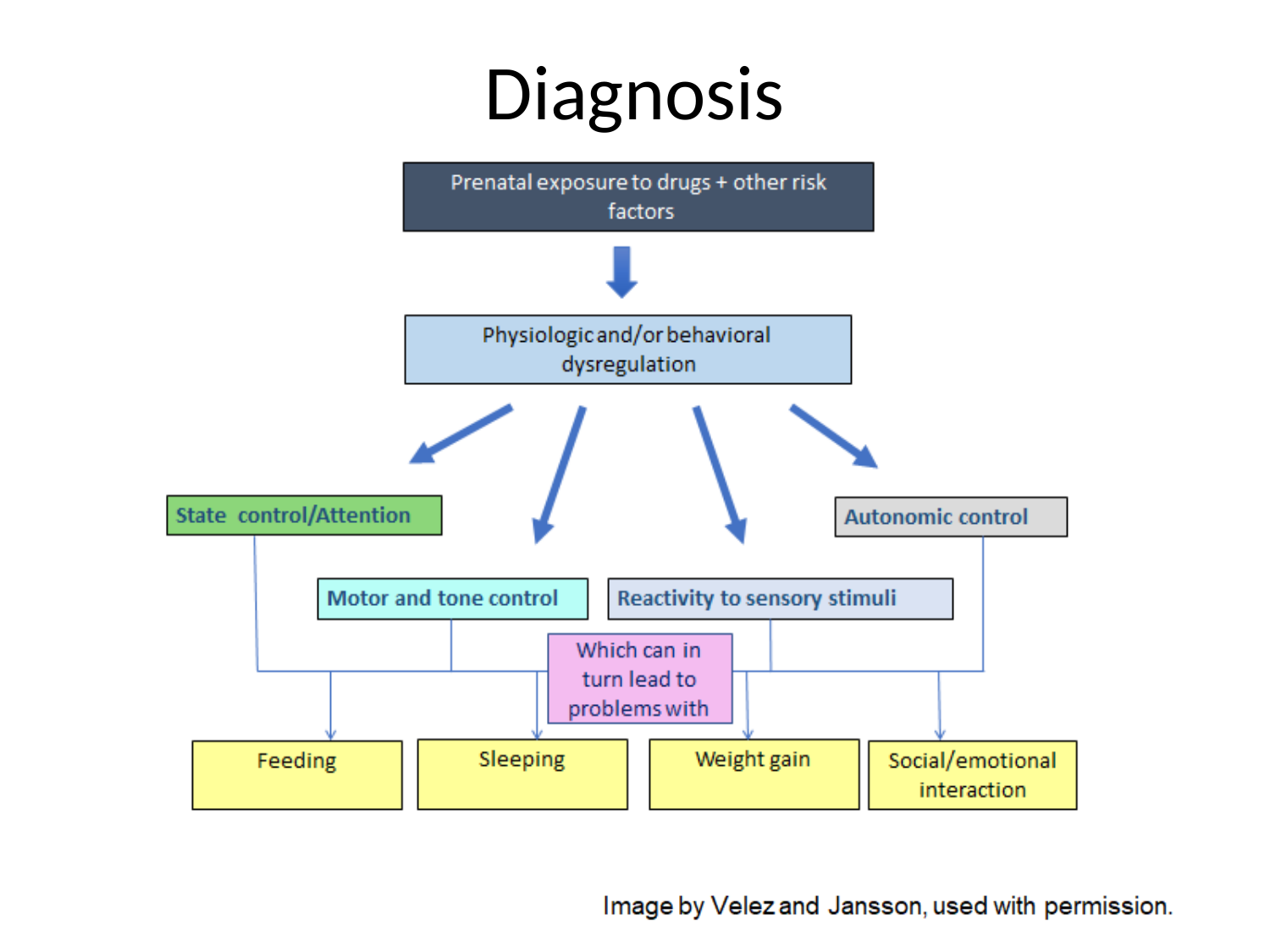

# Diagnosis

## Slide 10
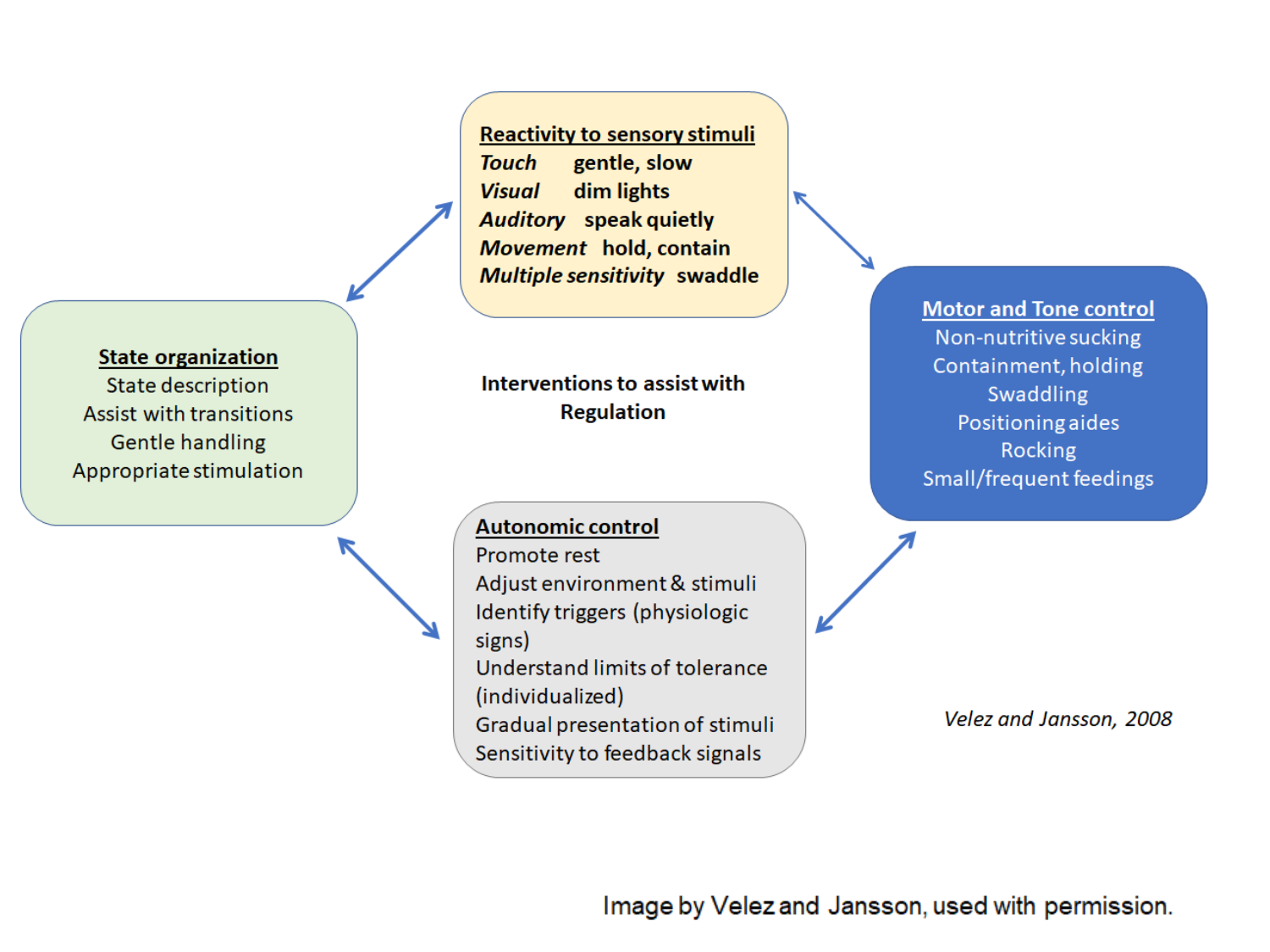

## Slide 11
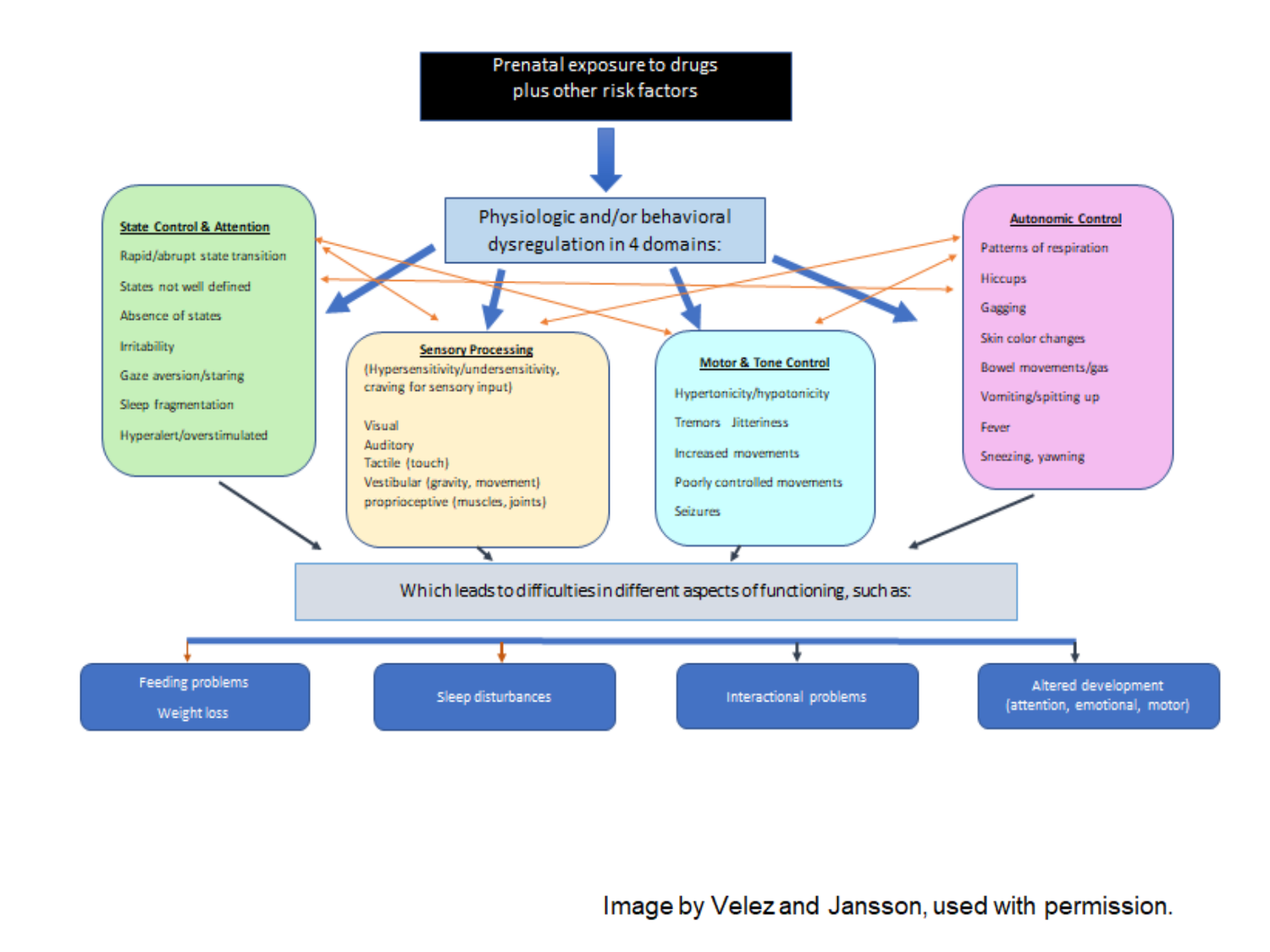

## Slide 12
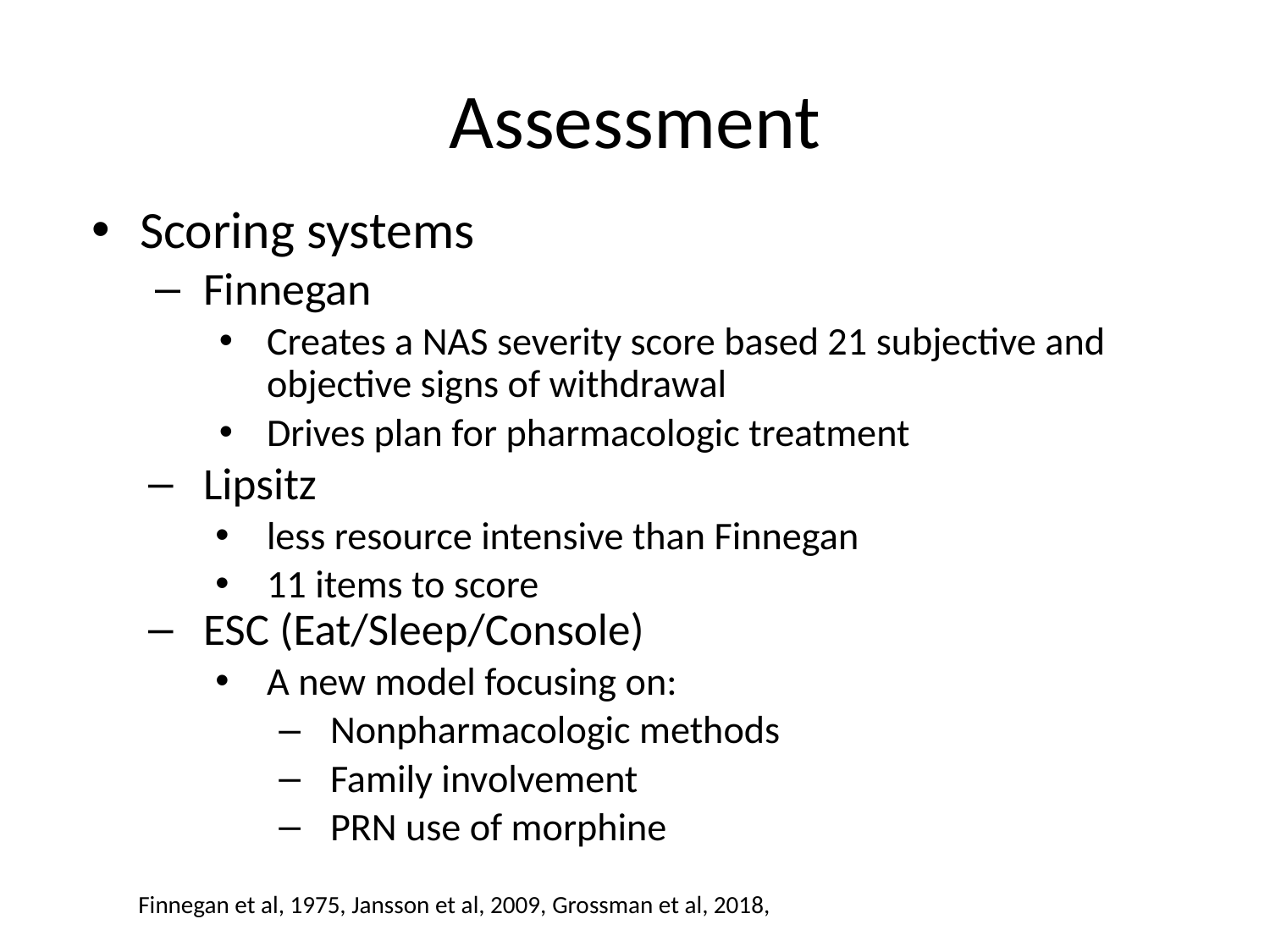

# Assessment
Scoring systems
Finnegan
Creates a NAS severity score based 21 subjective and objective signs of withdrawal
Drives plan for pharmacologic treatment
Lipsitz
less resource intensive than Finnegan
11 items to score
ESC (Eat/Sleep/Console)
A new model focusing on:
Nonpharmacologic methods
Family involvement
PRN use of morphine
Finnegan et al, 1975, Jansson et al, 2009, Grossman et al, 2018,

## Slide 13
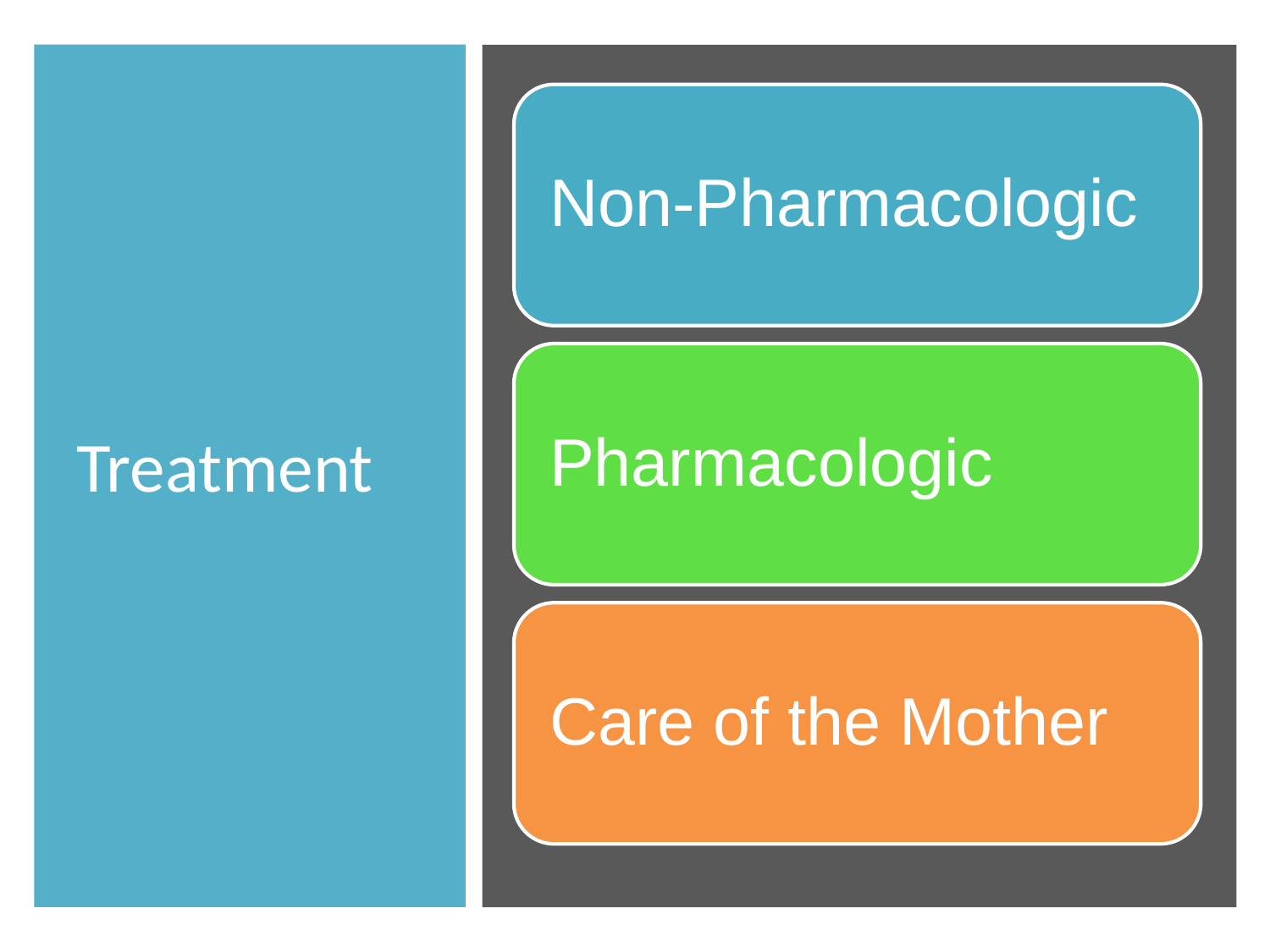

# Treatment
Non-Pharmacologic
Pharmacologic
Care of the Mother

## Slide 14
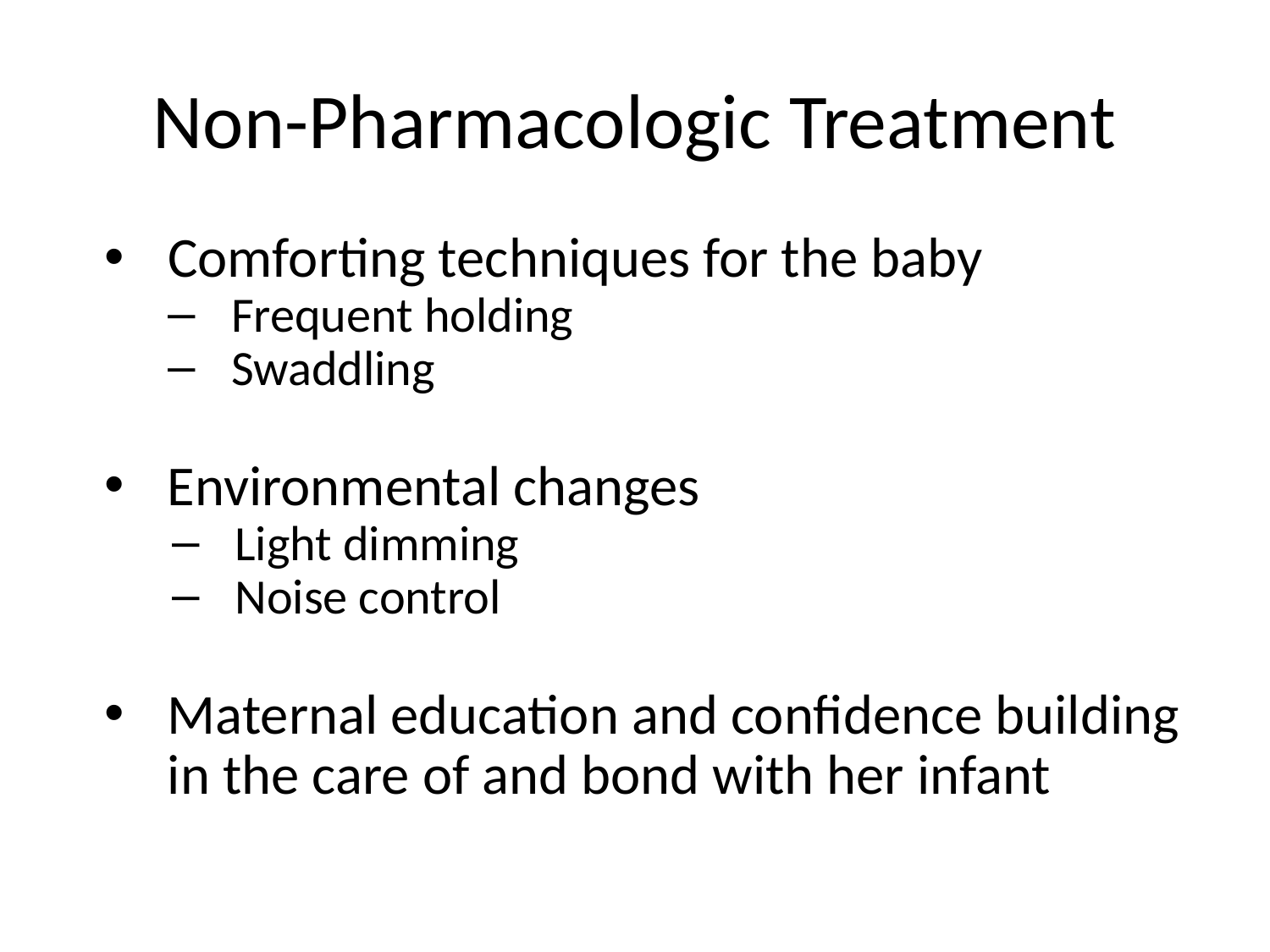

# Non-Pharmacologic Treatment
Comforting techniques for the baby
Frequent holding
Swaddling
Environmental changes
Light dimming
Noise control
Maternal education and confidence building in the care of and bond with her infant

## Slide 15
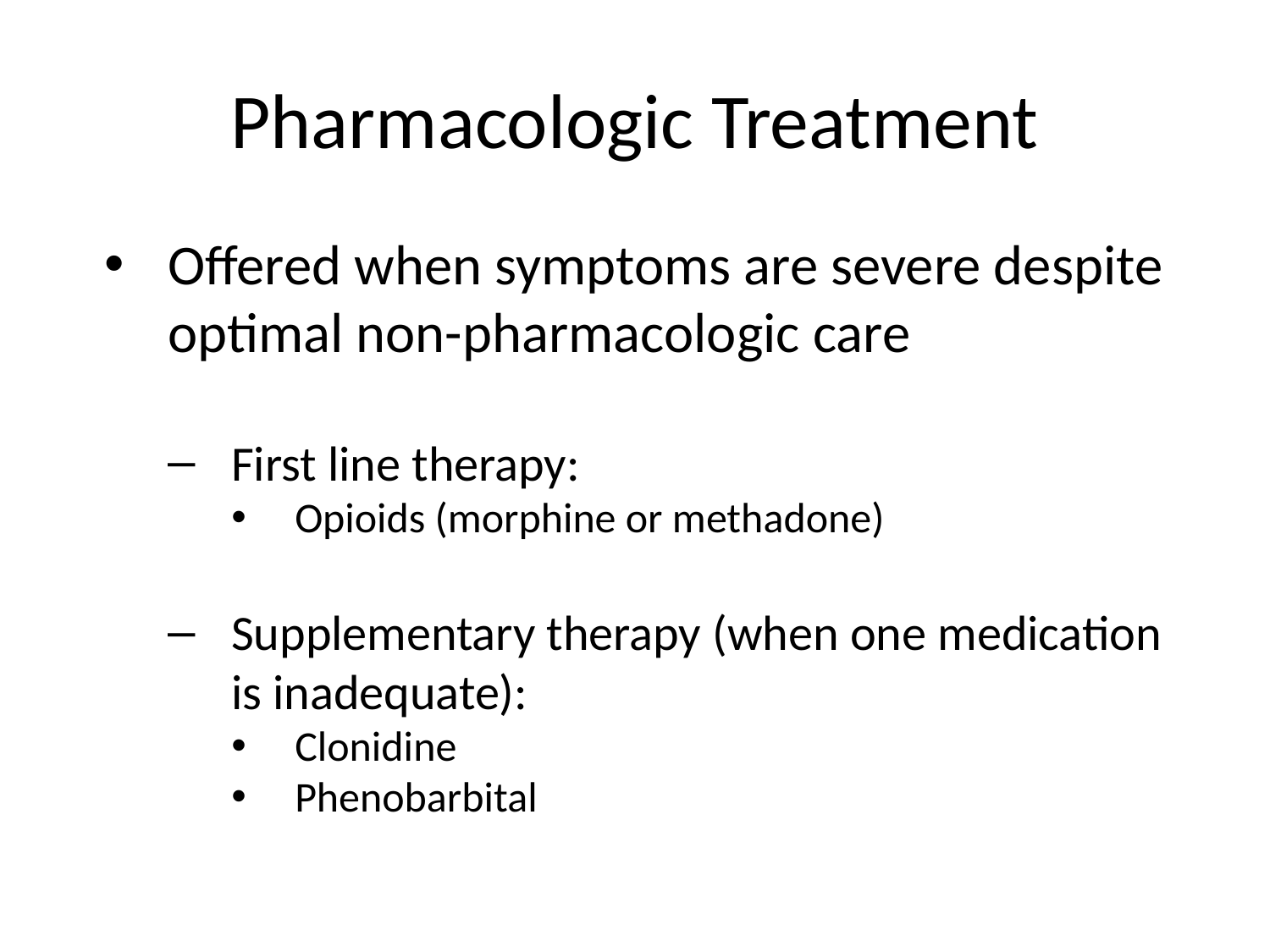

# Pharmacologic Treatment
Offered when symptoms are severe despite optimal non-pharmacologic care
First line therapy:
Opioids (morphine or methadone)
Supplementary therapy (when one medication is inadequate):
Clonidine
Phenobarbital

## Slide 16
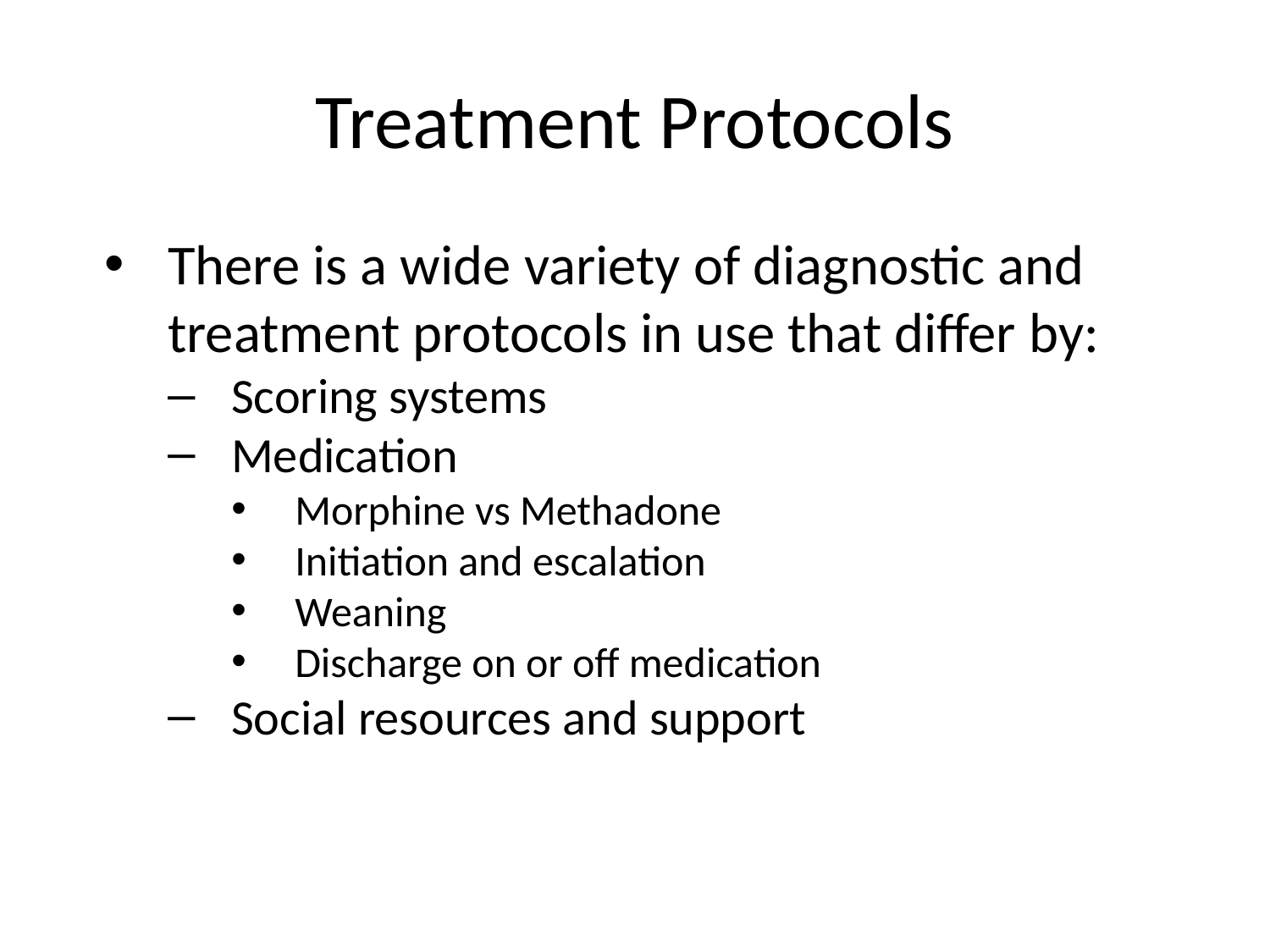

# Treatment Protocols
There is a wide variety of diagnostic and treatment protocols in use that differ by:
Scoring systems
Medication
Morphine vs Methadone
Initiation and escalation
Weaning
Discharge on or off medication
Social resources and support

## Slide 17
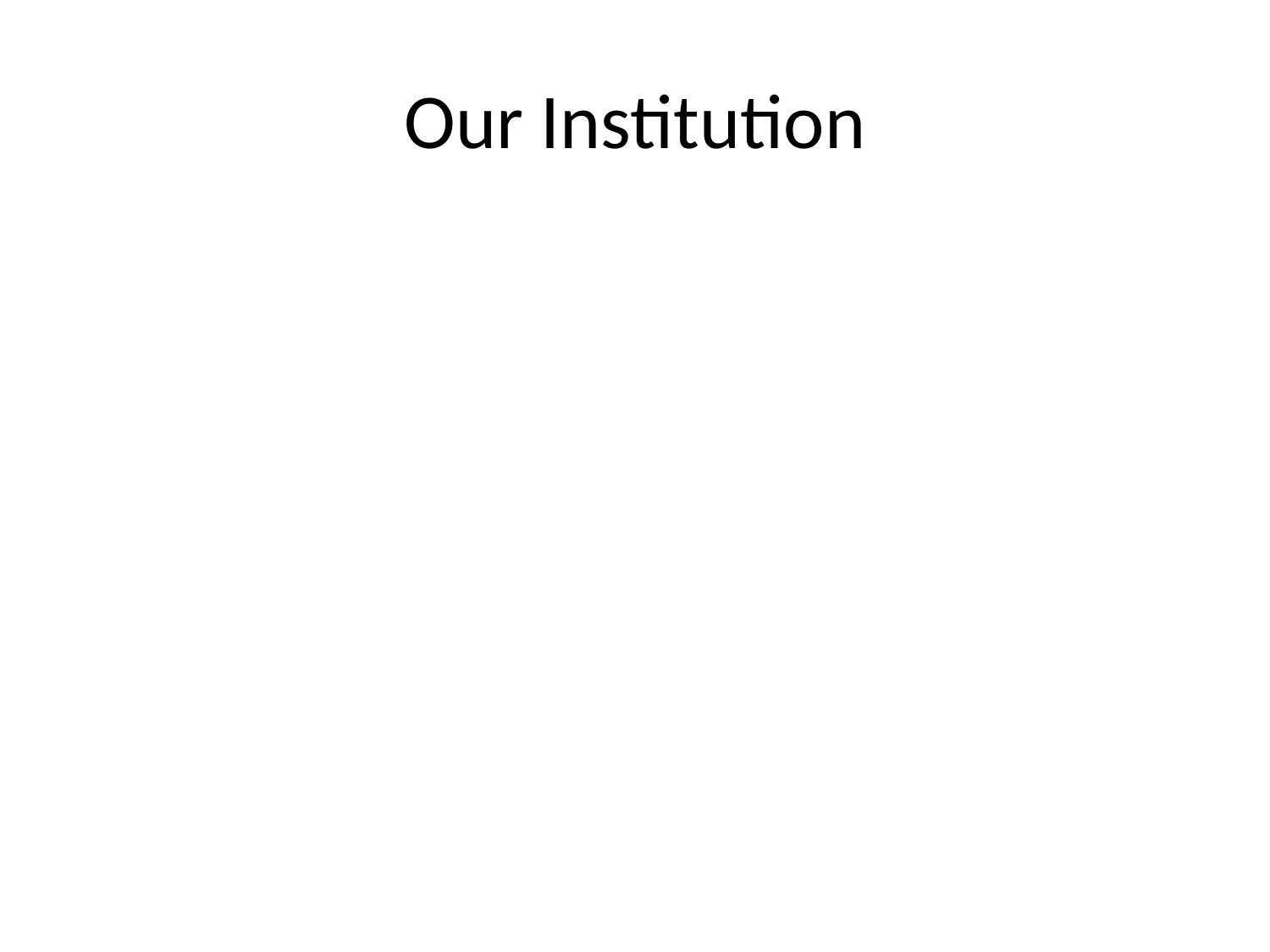

# Our Institution

## Slide 18
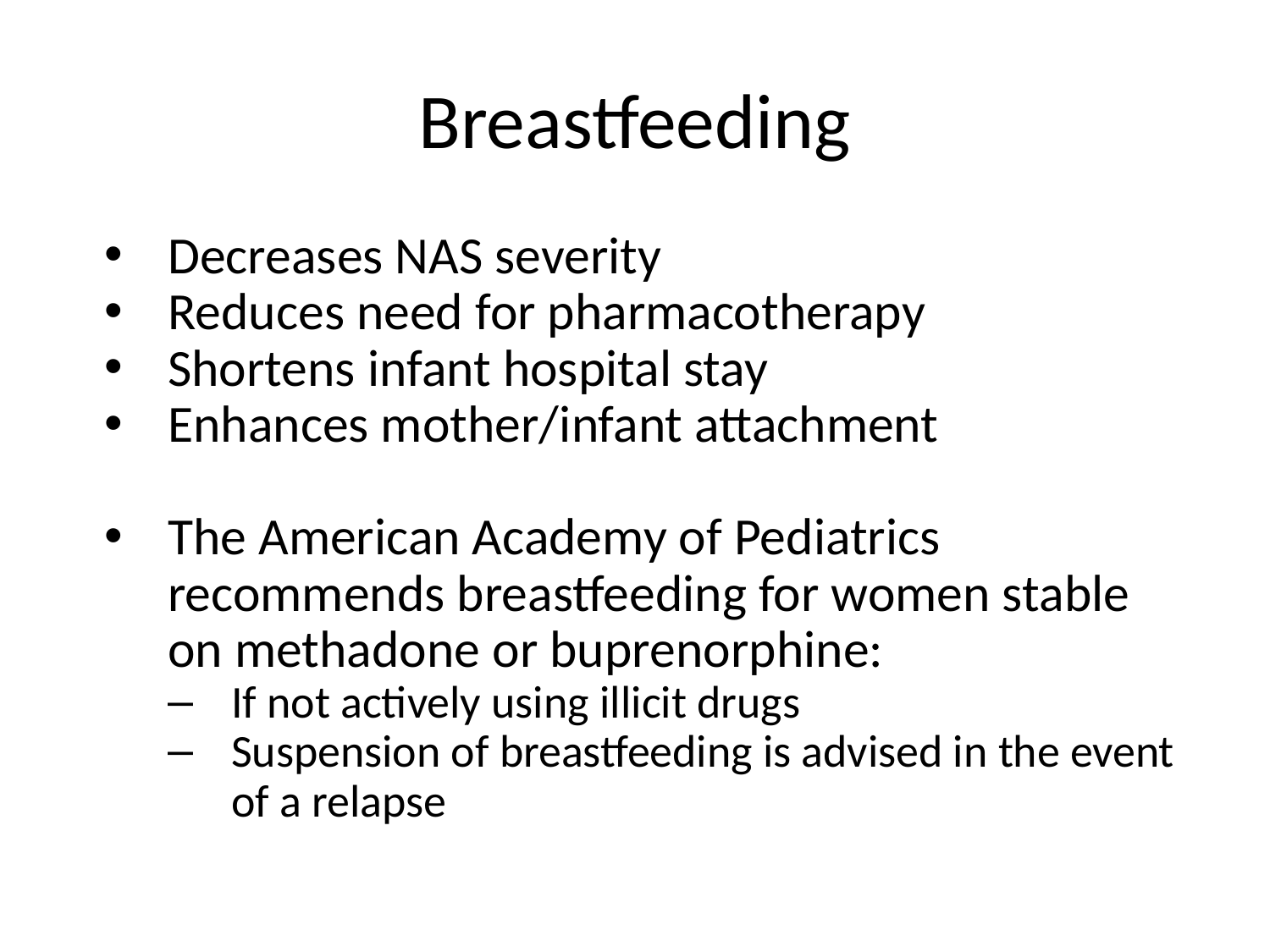

# Breastfeeding
Decreases NAS severity
Reduces need for pharmacotherapy
Shortens infant hospital stay
Enhances mother/infant attachment
The American Academy of Pediatrics recommends breastfeeding for women stable on methadone or buprenorphine:
If not actively using illicit drugs
Suspension of breastfeeding is advised in the event of a relapse

## Slide 19
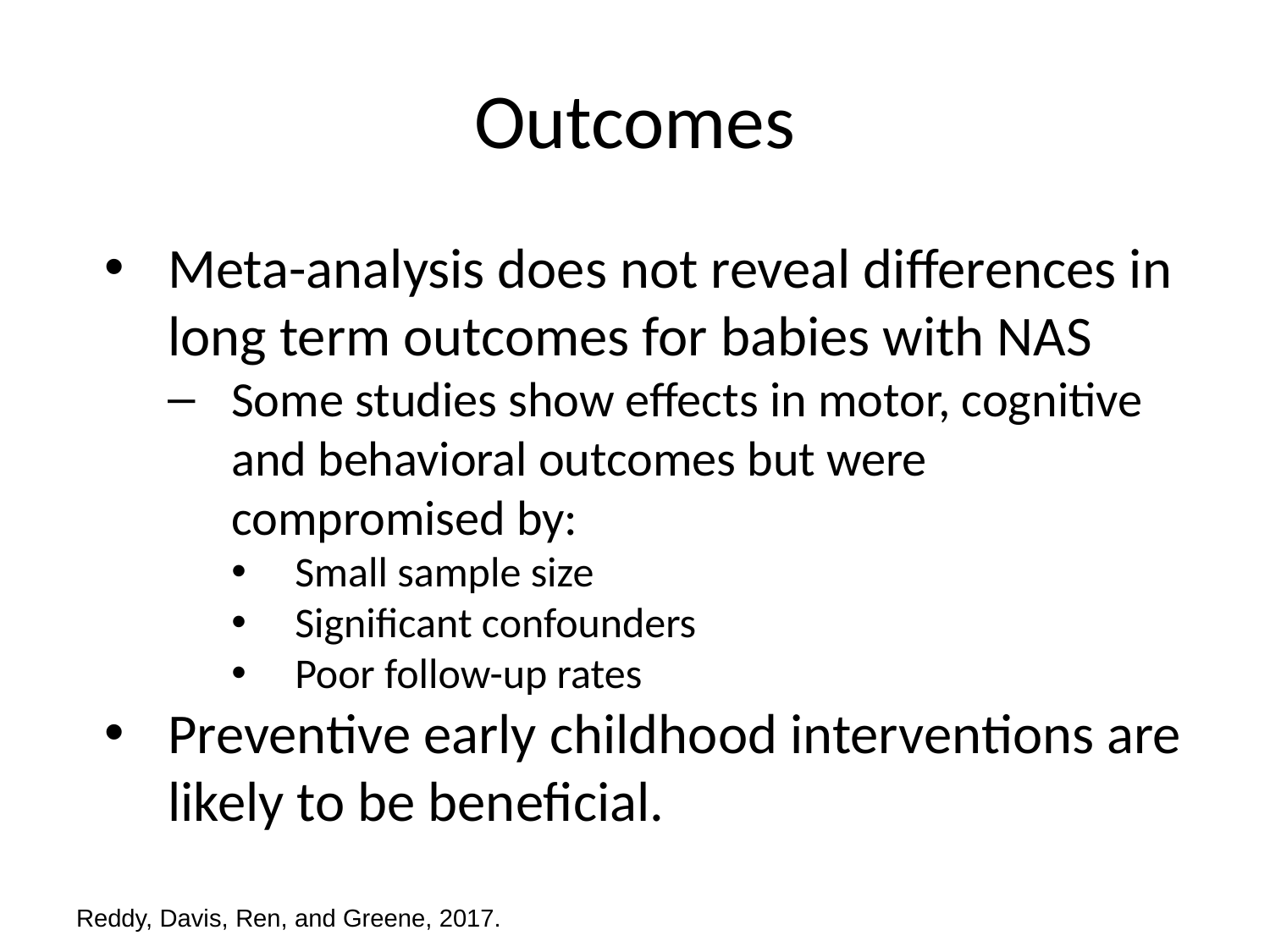

# Outcomes
Meta-analysis does not reveal differences in long term outcomes for babies with NAS
Some studies show effects in motor, cognitive and behavioral outcomes but were compromised by:
Small sample size
Significant confounders
Poor follow-up rates
Preventive early childhood interventions are likely to be beneficial.
Reddy, Davis, Ren, and Greene, 2017.

## Slide 20
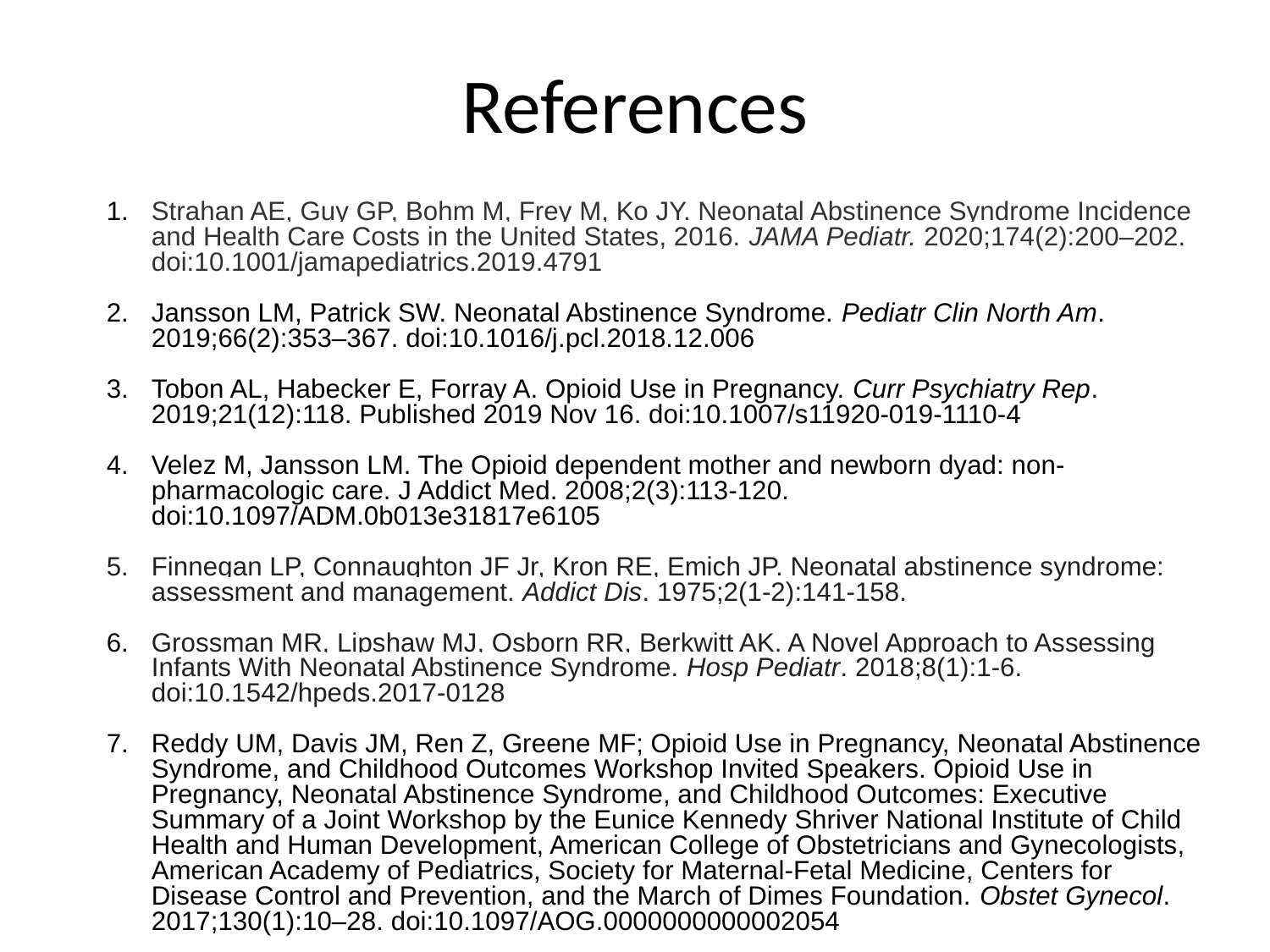

# References
Strahan AE, Guy GP, Bohm M, Frey M, Ko JY. Neonatal Abstinence Syndrome Incidence and Health Care Costs in the United States, 2016. JAMA Pediatr. 2020;174(2):200–202. doi:10.1001/jamapediatrics.2019.4791
Jansson LM, Patrick SW. Neonatal Abstinence Syndrome. Pediatr Clin North Am. 2019;66(2):353–367. doi:10.1016/j.pcl.2018.12.006
Tobon AL, Habecker E, Forray A. Opioid Use in Pregnancy. Curr Psychiatry Rep. 2019;21(12):118. Published 2019 Nov 16. doi:10.1007/s11920-019-1110-4
Velez M, Jansson LM. The Opioid dependent mother and newborn dyad: non-pharmacologic care. J Addict Med. 2008;2(3):113‐120. doi:10.1097/ADM.0b013e31817e6105
Finnegan LP, Connaughton JF Jr, Kron RE, Emich JP. Neonatal abstinence syndrome: assessment and management. Addict Dis. 1975;2(1-2):141‐158.
Grossman MR, Lipshaw MJ, Osborn RR, Berkwitt AK. A Novel Approach to Assessing Infants With Neonatal Abstinence Syndrome. Hosp Pediatr. 2018;8(1):1‐6. doi:10.1542/hpeds.2017-0128
Reddy UM, Davis JM, Ren Z, Greene MF; Opioid Use in Pregnancy, Neonatal Abstinence Syndrome, and Childhood Outcomes Workshop Invited Speakers. Opioid Use in Pregnancy, Neonatal Abstinence Syndrome, and Childhood Outcomes: Executive Summary of a Joint Workshop by the Eunice Kennedy Shriver National Institute of Child Health and Human Development, American College of Obstetricians and Gynecologists, American Academy of Pediatrics, Society for Maternal-Fetal Medicine, Centers for Disease Control and Prevention, and the March of Dimes Foundation. Obstet Gynecol. 2017;130(1):10–28. doi:10.1097/AOG.0000000000002054
