# Supplementary material for: The NICU Cuddler Curriculum: A Service-Learning Curriculum for Preclinical Medical Students in the Neonatal Intensive Care Unit
Source: MedEdPORTAL. 2021 Jan 12;17:11069. doi: 10.15766/mep_2374-8265.11069 (PMC7809928; doi:10.15766/mep_2374-8265.11069)

This log is intended to track who has cuddled and which patients have been cuddled, as useful information for later sessions, as well as for safety and QI purposes.

1. Log your activities during each cuddling shift

a. Please document whether you left a note at the bedside

b. Please document any notable events or helpful information for other cuddlers (ie, the baby had a lot of emesis, remember to wear a waterproof gown)

c. Return this log to its original secure location


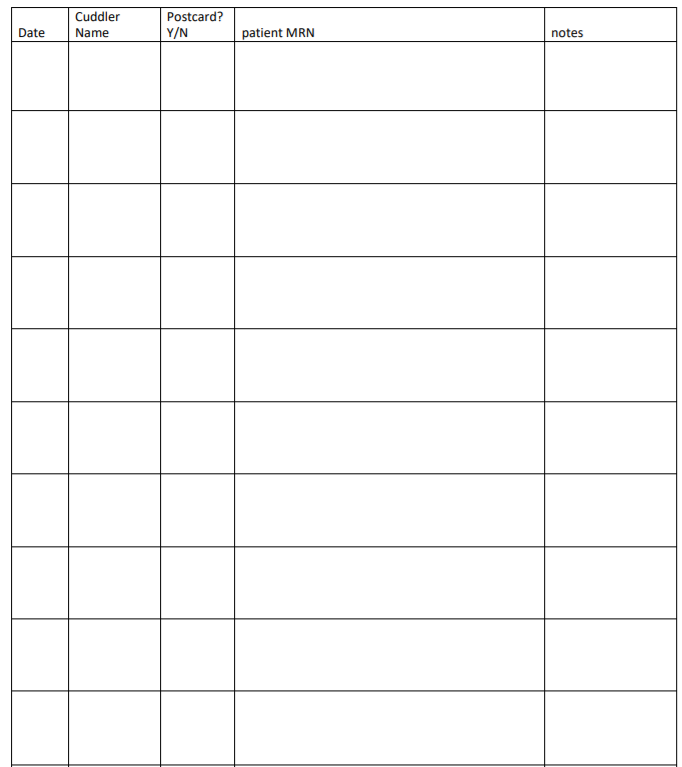

Supplement: Supplementary file 1 — Course Description.docxParticipant Application.docxOrientation Outline.docxOrientation Presentation.pptxNeonatal Abstinence Syndrome.pptxDevelopmental Care in the NICU.pptxParent Note Cards.docxPatient Log.docxAnonymous Concerns.docxStudent Survey.docxThird- and Fourth-Year Student Survey.docxEmail to Nursing Staff.docx [file mep_2374-8265.11069-s001.zip › H. Patient Log.docx]
