# Supplementary material for: The NICU Cuddler Curriculum: A Service-Learning Curriculum for Preclinical Medical Students in the Neonatal Intensive Care Unit
Source: MedEdPORTAL. 2021 Jan 12;17:11069. doi: 10.15766/mep_2374-8265.11069 (PMC7809928; doi:10.15766/mep_2374-8265.11069)
Supplement: Supplementary file 1 — Course Description.docxParticipant Application.docxOrientation Outline.docxOrientation Presentation.pptxNeonatal Abstinence Syndrome.pptxDevelopmental Care in the NICU.pptxParent Note Cards.docxPatient Log.docxAnonymous Concerns.docxStudent Survey.docxThird- and Fourth-Year Student Survey.docxEmail to Nursing Staff.docx [file mep_2374-8265.11069-s001.zip › I. Anonymous Concerns.docx]

Use: We used a Google Form for this information. An email with the form was sent out periodically to the student participants.

**Anonymous Concerns/Questions**

This is a safe space to share experiences, concerns, questions or anything else that may arise during shifts. Only the Cuddling leaders will see these entries and they will remain anonymous unless you would like to be contacted for further discussion. If this is the case, please leave your name as well.

1. Please share any concerns, questions, experiences or ideas with the leadership team
2. I would like to be contacted regarding this entry (Yes/No)
3. If you selected Yes above, please enter your name
