# Supplementary material for: The NICU Cuddler Curriculum: A Service-Learning Curriculum for Preclinical Medical Students in the Neonatal Intensive Care Unit
Source: MedEdPORTAL. 2021 Jan 12;17:11069. doi: 10.15766/mep_2374-8265.11069 (PMC7809928; doi:10.15766/mep_2374-8265.11069)
Supplement: Supplementary file 1 — Course Description.docxParticipant Application.docxOrientation Outline.docxOrientation Presentation.pptxNeonatal Abstinence Syndrome.pptxDevelopmental Care in the NICU.pptxParent Note Cards.docxPatient Log.docxAnonymous Concerns.docxStudent Survey.docxThird- and Fourth-Year Student Survey.docxEmail to Nursing Staff.docx [file mep_2374-8265.11069-s001.zip › J. Student Survey.docx]

Use: The following survey was administered to the student participants using Qualtrics.

**NICU Cuddler Participation Survey**

Please answer the following questions about your year spent in the NICU Cuddler Curriculum

Student Name:

1. How do you identify? Circle One: Male Female Other
2. What year in medical school are you? Circle One: MS1 MS2 MS3 MS4
3. Prior to participation in this program, had you ever held a newborn before? Circle One: Yes No
4. What areas of medicine are you interested in?

______________________________________________________________________________________

1. After participating, what purpose do you believe the NICU Cuddler Service Learning Program serves? Is it different than your preconception of the program before participation?

______________________________________________________________________________________________________________________________________________________________________________________________________________________________________________________________________________________________________________________________________________________________________________________________________________________________________________

1. Was your participation in this program useful to you? In what ways?

________________________________________________________________________________________________________________________________________________________________________________________________________________________________________________________________________________________________________________________________________________________
______________________________________________________________________________________

1. Did your participation in this program change your approach with patients? If it did, how?

______________________________________________________________________________________________________________________________________________________________________________________________________________________________________________________________________________________________________________________________________________________________________________________________________________________________________________

1. Reflecting on your experience, was there a specific memorable moment? Why did this moment stand out in your mind?

____________________________________________________________________________________________________________________________________________________________________________________________________________________________________________________________________________________________________________________________________________________________________________________________________________________________________________________________________________________________________________________________________

1. Did you gain any new skills from participating in this program? What were they?

______________________________________________________________________________________________________________________________________________________________________________________________________________________________________________________________________________________________________________________________________________________________________________________________________________________________________________

1. From your perspective, do families value the program? Support Staff (nurses)? In what ways?

______________________________________________________________________________________________________________________________________________________________________________________________________________________________________________________________________________________________________________________________________________________________________________________________________________________________________________

1. How would you improve the program?

______________________________________________________________________________________________________________________________________________________________________________________________________________________________________________________________________________________________________________________________________________________________________________________________________________________________________________

1. Would you recommend this program to other classmates?

______________________________________________________________________________________
