# Supplementary material for: The NICU Cuddler Curriculum: A Service-Learning Curriculum for Preclinical Medical Students in the Neonatal Intensive Care Unit
Source: MedEdPORTAL. 2021 Jan 12;17:11069. doi: 10.15766/mep_2374-8265.11069 (PMC7809928; doi:10.15766/mep_2374-8265.11069)
Supplement: Supplementary file 1 — Course Description.docxParticipant Application.docxOrientation Outline.docxOrientation Presentation.pptxNeonatal Abstinence Syndrome.pptxDevelopmental Care in the NICU.pptxParent Note Cards.docxPatient Log.docxAnonymous Concerns.docxStudent Survey.docxThird- and Fourth-Year Student Survey.docxEmail to Nursing Staff.docx [file mep_2374-8265.11069-s001.zip › K. Third- and Fourth-Year Student Survey.docx]

Use: The following survey was administered to the student participants using ___________.

**NICU Cuddler 3rd and 4th Year Survey**

Please answer the following questions about your time spent in the NICU Cuddler Curriculum

What year in medical school are you? Circle One: MS3 MS4

1. Being a NICU Cuddler helped me better understand risk factors for NICU Admission including clinical conditions and social determinants of health.

- Strongly Disagree
- Disagree
- Neutral
- Agree
- Strongly Agree

1. Participating in the NICU Cuddlers made me more confident in my ability to communicate effectively and work with an interdisciplinary team.

- Strongly Disagree
- Disagree
- Neutral
- Agree
- Strongly Agree

1. NICU Cuddling increased my confidence with cuddling techniques such as holding and transferring a baby.

- Strongly Disagree
- Disagree
- Neutral
- Agree
- Strongly Agree

1. Participating in NICU Cuddlers increased my understanding and ability to recognize social and emotional stressors faced by families of NICU patients.

- Strongly Disagree
- Disagree
- Neutral
- Agree
- Strongly Agree

1. Participating in NICU Cuddlers during my preclinical years provided me with transferable skills that I have used during my clinical rotations in 3rd and/or 4th year.

- Strongly Disagree
- Disagree
- Neutral
- Agree
- Strongly Agree

1. In what ways, if any, did the NICU Cuddler Curriculum prepare you for clinical rotations?

______________________________________________________________________________________________________________________________________________________________________________________________________________________________________________________________________________________________________________________________________________________________________________________________________________________________________________

____________________________________________________________________________________________________________________________________________________________________________

1. In retrospect, what could theNICU Cuddler Curriculum improve upon?

______________________________________________________________________________________________________________________________________________________________________________________________________________________________________________________________________________________________________________________________________________________________________________________________________________________________________________

____________________________________________________________________________________________________________________________________________________________________________

1. Other comments:

______________________________________________________________________________________________________________________________________________________________________________________________________________________________________________________________________________________________________________________________________________________________________________________________________________________________________________

____________________________________________________________________________________________________________________________________________________________________________
