# Supplementary material for: The NICU Cuddler Curriculum: A Service-Learning Curriculum for Preclinical Medical Students in the Neonatal Intensive Care Unit
Source: MedEdPORTAL. 2021 Jan 12;17:11069. doi: 10.15766/mep_2374-8265.11069 (PMC7809928; doi:10.15766/mep_2374-8265.11069)
Supplement: Supplementary file 1 — Course Description.docxParticipant Application.docxOrientation Outline.docxOrientation Presentation.pptxNeonatal Abstinence Syndrome.pptxDevelopmental Care in the NICU.pptxParent Note Cards.docxPatient Log.docxAnonymous Concerns.docxStudent Survey.docxThird- and Fourth-Year Student Survey.docxEmail to Nursing Staff.docx [file mep_2374-8265.11069-s001.zip › L. Email to Nursing Staff.docx]

***Subject: NICU Cuddler Program***

**[Hospital or Department Name]** are collaborating to launch a new program for medical students. The student participants have completed a 2-hour in-service for their Cuddler role in the NICU and will be able to hold and console our eligible NICU patients.

By providing a standardized classroom and hands-on orientation, the training for the students will allow them to more effectively communicate, understand their positive impact on NICU patients, and gain exposure to the NICU environment before starting clinical rotations. You will begin seeing them **[insert your institutions Cuddling dates and times]** as we pilot the program. In addition to their Cuddler time, they will be completing additional education offerings that relate to the NICU and related research. If you have questions or feedback on the program, please contact **[Insert Faculty Supervisors]**. We will work to get their pictures to nursing stations, so you get to know them over the coming months.
