# Supplementary figures and images for: The NICU Cuddler Curriculum: A Service-Learning Curriculum for Preclinical Medical Students in the Neonatal Intensive Care Unit
Source: MedEdPORTAL. 2021 Jan 12;17:11069. doi: 10.15766/mep_2374-8265.11069 (PMC7809928; doi:10.15766/mep_2374-8265.11069)

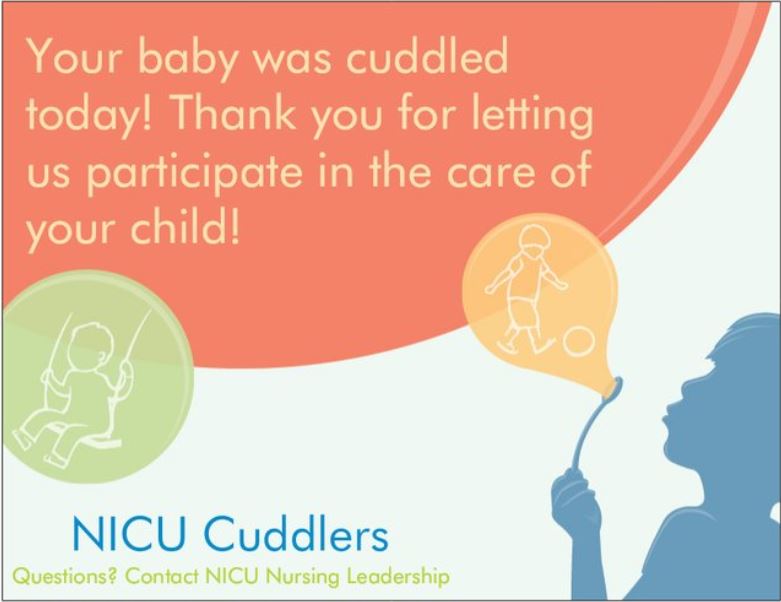


**Side A**


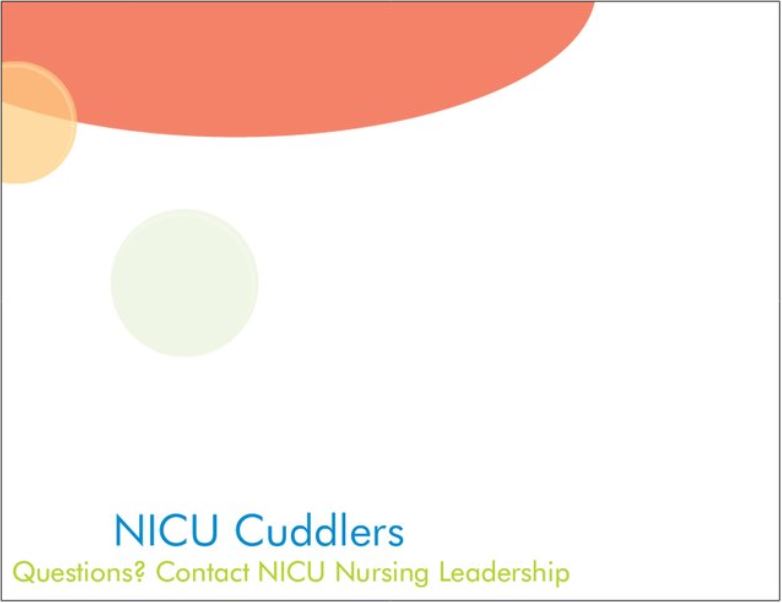


**Side B**

Supplement: Supplementary file 1 — Course Description.docxParticipant Application.docxOrientation Outline.docxOrientation Presentation.pptxNeonatal Abstinence Syndrome.pptxDevelopmental Care in the NICU.pptxParent Note Cards.docxPatient Log.docxAnonymous Concerns.docxStudent Survey.docxThird- and Fourth-Year Student Survey.docxEmail to Nursing Staff.docx [file mep_2374-8265.11069-s001.zip › G. Parent Note Cards.docx]
